# Sex-Dependent Changes in Quality and Flavor-Related Chemical Profiles of *Mytilus coruscus* During Long-Term Frozen Storage

**DOI:** 10.3390/foods15183351

**Published:** 2026-09-21

**Authors:** Ruoshu Li, Xiaojian Xiu, Jiangbo Xiang, Kai Xu, Jixin Luo, Yaqing Wang, Xiaoming Jiang, Ye Liu, Tingyu Feng, Changhu Xue

**Affiliations:** 1 College of Food Science & Engineering, Ocean University of China, Qingdao 266404, China; 2 Qingdao Institute of Marine Bioresources for Nutrition & Health Innovation, Qingdao 266109, China; 3 China Resources Wind Power (Shanwei) Co., Ltd., Shanwei 516600, China; 4 Quanzhou Institute of Marine Bioresources Industry, Quanzhou 362700, China

**Keywords:** cultivated mussel, sex differences, frozen storage, non-volatile compounds, GC-IMS, fatty acid composition

## Abstract

The effects of frozen storage on the quality and flavor of female and male Mytilus coruscus were investigated at −18 °C for 0, 90, and 150 days through analyses of physicochemical properties, free amino acids, 5′-nucleotides, fatty acids, and volatile organic compounds. Distinct sex-dependent changes were observed in the quality and flavor profiles. After 150 days, the centrifugal loss reached 1.2 and 1.5 times the fresh levels in females and males, respectively, whereas total volatile basic nitrogen reached 2.7 and 2.4 times the corresponding fresh levels. Malondialdehyde increased to approximately 3.3 times the fresh level in both sexes and was 1.7 times as high in females as in males. Males generally showed higher free amino acid levels, whereas females showed higher equivalent umami concentrations at selected stages. Gas chromatography-ion mobility spectrometry revealed sex-dependent volatile organic compound (VOC) profiles. Aldehydes were enriched in females after 90 days, whereas males retained more pronounced ketone- and ester-related characteristics. Fourteen key discriminative VOCs were selected. Correlation analysis linked changes in VOC profiles with nucleotide degradation, amino acid changes, fatty acid composition, and lipid oxidation; notably, 1-penten-3-one was positively correlated with malondialdehyde and negatively correlated with eicosapentaenoic acid (EPA), docosahexaenoic acid (DHA), and arachidonic acid (ARA). These associations provide new insights into sex-specific changes in flavor-related chemical profiles during frozen storage and provide a scientific basis for improving quality evaluation and frozen-storage strategies for mussels in the seafood industry.

## 1. Introduction

Thick-shell mussel (*Mytilus coruscus*) is an economically important mariculture bivalve with high commercial and nutritional value and is widely cultivated along the coast of China [1]. According to the China Fishery Statistical Yearbook 2026 [2], the marine aquaculture production of mussels in China reached 780,900 t in 2025. Fresh mussels are appreciated for their characteristic flavor and texture but are highly perishable during postharvest storage, which limits their shelf life, distribution, and marketability. Freezing is widely used for the long-term preservation of aquatic products because it effectively inhibits microbial growth and slows enzymatic reactions [3,4]. Owing to their high moisture content and delicate tissue structure, mussels are particularly susceptible to quality deterioration during prolonged frozen storage, which can induce water migration, protein degradation, lipid oxidation, and ice crystal-induced structural damage, ultimately reducing their water-holding capacity and causing textural deterioration and changes in flavor-related chemical profiles [5,6,7,8].

Flavor is an important quality attribute that influences consumer acceptance and sensory perception [9]. Aroma is primarily associated with volatile organic compounds (VOCs), whereas taste is mainly determined by free amino acids (FAAs), organic acids, and 5′-nucleotides [9,10]. Volatile and non-volatile flavor components are closely interconnected because amino acids, fatty acids, and other flavor precursors can undergo degradation or oxidation during processing and storage, thereby contributing to VOC formation. The recovery of residual enzyme activity during thawing may further promote the transformation of proteins, 5′-nucleotides, and other components [11]. In particular, the degradation of proteins and small peptides can alter FAA levels, whereas the conversion of adenosine triphosphate (ATP) and its related compounds can modify the composition of adenosine 5′-monophosphate (5′-AMP), inosine 5′-monophosphate (5′-IMP), and other taste-active nucleotides [12]. Meanwhile, unsaturated fatty acids are susceptible to oxidation, generating low-molecular-weight volatile compounds such as aldehydes, ketones, and alcohols, some of which contribute to rancid odors [13]. Degradation of amino acids and other nitrogen- or sulfur-containing compounds may also generate amines, ammonia, and sulfur-containing volatiles, further modifying the overall flavor profile [5]. Thus, flavor evolution during frozen storage reflects the combined effects of protein degradation, nucleotide conversion, lipid oxidation, and related biochemical reactions, with subsequent consequences for sensory quality and consumer acceptance [14].

Previous studies on frozen aquatic products have characterized quality changes using physicochemical attributes and flavor-related indicators, including water-holding properties, texture, taste compounds, and volatile profiles [12,13,15,16]. However, these indicators are often investigated separately, and the coordinated relationships among free amino acids, taste-active 5′-nucleotides, fatty acid composition, and VOCs during frozen storage remain insufficiently understood. An integrated analysis of non-volatile taste compounds, fatty acid composition, and VOC is therefore required to more comprehensively characterize flavor evolution during frozen storage. Gas chromatography-ion mobility spectrometry (GC-IMS), characterized by rapid analysis, high sensitivity, and visual fingerprinting, has been widely applied to VOC analysis in aquatic products and is particularly suitable for monitoring changes in volatile flavor profiles during storage [17].

In addition to storage duration, sex-related biological differences in the raw material may also influence flavor responses during frozen storage. Mussels have separate sexes, and during the reproductive period, males and females can be readily distinguished by differences in gonadal coloration and sensory characteristics. Sex-related differences have also been reported in reproductive investment, lipid reserves, protein composition, and energy metabolism [18,19,20,21]. Zhang et al. reported significant differences in lipid and fatty acid composition between female and male *Mytilus edulis*, with males exhibiting a higher combined proportion of eicosapentaenoic acid (EPA) and docosahexaenoic acid (DHA) [19]. Zhao et al. further demonstrated that sex affected the formation of volatile compounds and metabolites during the thermal processing of mussels [18], while Ma et al. showed that sex significantly influenced lipid reserves and the tissue distribution of nutrients [22]. Collectively, these findings indicate that females and males differ in nutrient reserves, metabolic regulation, the conversion of 5′-nucleotides, and the formation of lipid oxidation products during frozen storage [23,24]. However, systematic comparisons of sex-dependent changes in non-volatile taste compounds, fatty acid composition, and VOC profiles of mussels during frozen storage remain limited.

Therefore, this study investigated female and male *Mytilus coruscus* during frozen storage at −18 °C for 0, 90, and 150 days, representing the fresh, intermediate, and prolonged storage stages, respectively. Changes in free amino acids, 5′-nucleotides, equivalent umami concentration (EUC), and fatty acid composition were determined, while GC-IMS combined with multivariate statistical analysis was used to characterize differences in VOCs. In parallel, changes in quality during frozen storage were evaluated using malondialdehyde (MDA), total volatile basic nitrogen (TVB-N), centrifugal loss, and texture properties. The study aimed to determine how storage duration and sex influence quality deterioration and the evolution of non-volatile taste and volatile flavor profiles in *M. coruscus* during long-term frozen storage.

## 2. Materials and Methods

### 2.1. Chemicals

High-performance liquid chromatography (HPLC)-grade solvents were purchased from Fisher Chemical (Fisher Scientific, Pittsburgh, PA, USA). The malondialdehyde (MDA) assay kit was obtained from the Nanjing Jiancheng Bioengineering Institute (Nanjing, China). Amino acid standards and the corresponding derivatization kit were obtained from Welch Materials Co., Ltd. (Shanghai, China). Nucleotide and organic acid standards were purchased from Tanmo Quality Inspection Technology Co., Ltd. (Beijing, China). Standard fatty acid methyl ester (FAME) mixtures were sourced from ANPEL Laboratory Technologies Inc. (Shanghai, China). Ultra-pure water was prepared using a Milli-Q water purification system (Millipore, Bedford, MA, USA). All the other chemicals and reagents used in the study were of analytical grade and were purchased from Sinopharm Chemical Reagent Co., Ltd. (Shanghai, China).

### 2.2. Sample Collection and Preparation

Live cultivated *M. coruscus* were collected from Rizhao, Shandong Province. Samples were collected in March 2025, approximately 2–3 years old. All mussels in the present study were collected during a single sampling period in March to maintain a consistent seasonal background and minimize the variability associated with seasonal changes. In our previous study using mussels from the same sampling batch, this period was associated with active gonadal development prior to the main spring spawning season, as characterized by the H&E staining of representative gonadal tissues [25].

The mussel samples were packed into cold boxes (4 °C) and transported to the laboratory, within approximately 2 h from the moment of packaging. Upon arrival, the mussel shells were thoroughly rinsed with seawater (salinity 28 PSU) to remove surface debris. Then, the mussels were selected, with a mean shell length, shell width, shell height, and body mass of 97.99 ± 3.96 mm, 45.99 ± 2.87 mm, 31.88 ± 1.70 mm, and 48.96 ± 6.36 g, respectively. The shells were opened and sex was determined macroscopically based on gonadal coloration: orange-yellow gonads were classified as female and milky-white gonads as male [26]. The soft tissues were removed from the shells, and the digestive tract and associated visceral tissues containing visible digestive contents were manually excised. The remaining soft tissues were used for subsequent analyses.

For each sex, 90 mussels were assigned to three storage times (0, 90, and 150 days), with 30 individuals per time point. Each group was randomly divided into three biological replicates of 10 individuals (*n* = 3). Each replicate was packed in a resealable polyethylene (PE) bag (10 cm × 20 cm; nominal thickness, 0.20 mm; Changde Beikeman Biotechnology Co., Ltd., Changde, China), with the mussels arranged in a single layer without overlapping. Of the 10 mussels in each replicate, two were allocated to physical measurements: one was first subjected to nondestructive color determination and subsequently used for texture analysis, whereas the other was used for centrifugal loss determination. The remaining eight mussels were pooled for chemical analyses.

The M0 and F0 groups were analyzed as fresh controls. For the M90, M150, F90, and F150 groups, samples were stored at −18 °C in the PE bags described above. After storage, the two mussels allocated to physical measurements were thawed at 4 °C for 6 h until the core temperature reached 4 °C, after which surface moisture was gently removed before analysis. For chemical analyses, the pooled tissues from the remaining eight mussels were cryogenically ground with liquid nitrogen using a grinder (MFJ-W317, Beijing Liven Technology Co., Ltd., Beijing, China) until homogeneous. The resulting samples were aliquoted into PE bags and stored at −80 °C until analysis.

### 2.3. Centrifugal Loss Content Determination

Centrifugal loss was determined following the method previously described [15], with minor adjustments. Briefly, the surface water of the thawed sample was removed and the sample was weighed (m_1_). Then, the sample was wrapped in filter paper and placed in a centrifuge tube with a layer of absorbent cotton at the bottom, and centrifuged at 3000 rpm for 15 min using a refrigerated centrifuge (TGL-16M, Hunan Xiangyi Laboratory Instrument Development Co., Ltd., Changsha, China). After centrifugation, the filter paper was removed and the sample was weighed (m_2_) again. The centrifugal loss was calculated using the following formula:

$$\mathrm{Centrifugal}\;\mathrm{loss}\;\left(\%\right)=\frac{m_{1}-m_{2}}{m_{1}}\times 100\%$$

### 2.4. Total Volatile Basic Nitrogen (TVB-N) Content Determination

The TVB-N values were determined in accordance with the Chinese Standard [27], with minor adjustments. A 3 g portion of the powdered sample was added to 20 mL of distilled water and thoroughly mixed. Subsequently, 1 g of magnesium oxide was added to the homogenized liquid for determination using a Kjeldahl nitrogen analyzer (K1160, Hanon, Jinan, China).

### 2.5. Malondialdehyde (MDA) Content Determination

The MDA content was determined using the thiobarbituric acid (TBA) method with a commercial assay kit (A003-1, Nanjing Jiancheng Bioengineering Institute, Nanjing, China) according to the manufacturer’s instructions. Briefly, mussel tissues were mixed with physiological saline at a tissue-to-saline ratio of 1:9 (*w*/*v*) to prepare a 10% (*w*/*v*) homogenate. The samples were homogenized in an ice-water bath using an ultrasonic cell disruptor (JY92-IIDN, Ningbo Scientz Biotechnology Co., Ltd., Ningbo, China). The homogenates were centrifuged at 3500 rpm for 10 min at 4 °C using a refrigerated centrifuge (TGL-16M), and the resulting supernatants were collected for analysis. The collected supernatants were mixed with the reagents provided in the assay kit according to the manufacturer’s instructions, heated in a water bath at 95 °C for 40 min, cooled in cold water, and centrifuged again at 3500 rpm for 10 min at 4 °C. The absorbance of the resulting supernatants was measured at 532 nm using a Multiskan SkyHigh spectrophotometer (Thermo Scientific, Life Technologies Holdings Pte Ltd., Singapore). The protein concentration in the supernatants was determined using the Coomassie brilliant blue (Bradford) method (A045-2, Nanjing Jiancheng Bioengineering Institute, Nanjing, China). The MDA content was calculated according to the manufacturer’s instructions and expressed as nmol/mg prot.

### 2.6. Color and Texture Determination

The color parameters of the mussel samples were measured using an RM200QC imaging spectrocolorimeter (X-Rite, Inc., Grand Rapids, MI, USA) [28]. Before measurement, the instrument was calibrated according to the manufacturer’s instructions. The CIE color parameters, including lightness (L_1_), redness/greenness (a_1_) and yellowness/blueness (b_1_), were recorded at different positions on the surface of each sample. Each sample was measured at least three times, and the average value was used for further analysis.

The total color difference (ΔE) was calculated to evaluate the overall color change during frozen storage according to the following equation:

$$∆E=\sqrt{{\left(L_{1}-L_{0}\right)}^{2}+{\left(a_{1}-a_{0}\right)}^{2}+{\left(b_{1}-b_{0}\right)}^{2}}$$

where L_1_, a_1_, and b_1_ represent the color parameters measured after frozen storage, and L_0_, a_0_, and b_0_ represent the corresponding initial values measured in female and male mussels at 0 d. For ΔE calculation, the fresh sample of each sex was used as its respective reference (F0 for females and M0 for males).

Texture profile analysis (TPA) was performed using a slightly modified method according to [15]. The trunk region of each thawed mussel sample was directly subjected to TPA using a texture analyzer (CT3, Brookfield Engineering Laboratories, Inc., Middleboro, MA, USA). A Sphere probe (TA43, 25.4 mm diameter) was employed in TPA mode. The measurement conditions were as follows: the test speed was 1 mm/min, there was 20% of the original sample height and the trigger force was 2 N.

### 2.7. Free Amino Acid Determination

Free amino acids (FAAs) were determined by HPLC following our previously reported method [25]. Briefly, homogenized mussel tissue was extracted with 5% trichloroacetic acid, and the extract was centrifuged at 10,000 rpm for 15 min. The resulting supernatant was derivatized using a Yuexu Amino Acid Derivatization Kit (Welch Materials Co., Ltd., Shanghai, China).and subsequently analyzed using an Agilent 1260 HPLC system (Agilent Technologies, Santa Clara, CA, USA) equipped with a variable wavelength detector (VWD) and an Ultimate Amino Acid column (4.6 mm × 250 mm, 5 μm; Welch Materials, Shanghai, China). Mobile phase A consisted of 0.1 mol/L sodium acetate buffer (pH 6.50)-acetonitrile (93:7, *v*/*v*), whereas mobile phase B consisted of acetonitrile-water (80:20, *v*/*v*). The flow rate was 1.00 mL/min, the column temperature was maintained at 40 °C, the injection volume was 10 μL, and detection was performed at 254 nm. Individual FAAs were identified by comparison of their retention times with those of authentic standards and quantified using the corresponding calibration curves.

Taurine content was determined with reference to Method II of GB 5009.169-2016 [27]. Briefly, mussel tissue was extracted with warm water, deproteinized with potassium ferrocyanide and zinc acetate, and derivatized with dansyl chloride. Taurine was analyzed using an Agilent 1260 HPLC system (Agilent Technologies, Santa Clara, CA, USA) equipped with a variable wavelength detector (VWD) and a ZORBAX Eclipse XDB-C18 column. The mobile phase consisted of 10 mmol/L sodium acetate buffer (pH 4.2)-acetonitrile (70:30, *v*/*v*) at a flow rate of 1.00 mL/min. Detection was performed at 254 nm, and taurine was quantified using an external calibration curve.

### 2.8. 5′-Nucleotide Determination

5′-Nucleotides were determined by HPLC according to our previously reported method [25]. An Agilent 1260 HPLC system equipped with a CAPCELL PAK C18 SG column (4.6 mm × 150 mm, 5 μm; Shiseido Co., Ltd., Tokyo, Japan) and a variable wavelength detector (VWD) was used. Isocratic separation was performed with an aqueous mobile phase containing 20 mmol/L phosphoric acid, 20 mmol/L citric acid, and 40 mmol/L triethylamine at a flow rate of 1.00 mL/min. Detection was conducted at 254 nm. Individual 5′-nucleotides were identified by comparison with the retention times of authentic standards and quantified using external calibration curves based on peak areas.

### 2.9. Equivalent Umami Concentration Determination

Equivalent umami concentration (EUC), expressed as g monosodium glutamate (MSG)/100 g, was calculated according to the previously described method [17]. EUC represents the synergistic umami intensity contributed by umami amino acids (Asp and Glu) and 5′-nucleotides (5′-IMP, 5′-GMP, and 5′-AMP). The calculation was performed using the following equation:

EUC (g MSG/100 g) = ∑a_i_b_i_ + 1218 (∑a_i_b_i_) (∑a_j_b_j_)

a_i_: UAA concentration (g/100 g) (Asp or Glu);
b_i_: UAA freshness coefficient relative to MSG (where Asp is 0.077 and Glu is 1.0);
a_j_: 5′-nucleotide concentration (g/100 g) (5′-IMP, 5′-GMP, 5′-AMP);
b_j_: taste nucleotide freshness coefficient relative to IMP (where 5′-IMP is 1.0, 5′-GMP is 2.3, and 5′-AMP is 0.18).

### 2.10. Fatty Acid Composition Determination

Fatty acid composition was determined according to GB 5009.168-2016 [29] and our previously reported procedure [25]. Briefly, lipids released by hydrochloric acid hydrolysis were extracted with petroleum ether-diethyl ether, followed by saponification with sodium hydroxide-methanol at 80 °C and methylation with boron trifluoride-methanol at 80 °C for 2 min. The resulting fatty acid methyl esters (FAMEs) were extracted with n-heptane and dehydrated with anhydrous sodium sulfate. FAMEs were analyzed using an Agilent 8890 gas chromatograph (Agilent Technologies, Santa Clara, CA, USA) equipped with a flame ionization detector (FID) and a DB-Fast FAME column (20 m × 0.18 mm, 0.20 μm). Samples were introduced through a split inlet operated at 250 °C with a split ratio of 100:1. The oven temperature was programmed from 80 °C to 220 °C at 25 °C/min, held for 3 min, and subsequently increased to 245 °C at 5 °C/min and held for 3 min. Nitrogen was used as the carrier gas, and the FID temperature was maintained at 250 °C. Fatty acids were identified using a standard FAME mixture and expressed as relative percentages based on peak-area normalization.

### 2.11. Gas Chromatography-Ion Mobility Spectrometry (GC-IMS) Determination

Volatile organic compound (VOC) profiles were analyzed by GC-IMS (FlavourSpec^®^, Gesellschaft für Analytische Sensorsysteme mbH, Dortmund, Germany) according to a previously described method [25]. Briefly, 2.00 g of cryogenically ground and thoroughly homogenized mussel tissue was accurately weighed into a 20 mL headspace vial. The sample was incubated at 60 °C for 10 min with agitation at 500 rpm prior to GC-IMS analysis. High-purity nitrogen was used as the carrier gas, with the flow rate programmed at 2 mL/min for 5 min, followed by 100 mL/min for 5 min and 150 mL/min for 7 min. VOCs were qualitatively identified by comparing their retention times (RTs) and drift times (DTs) with reference information in the National Institute of Standards and Technology (NIST) and ion mobility spectrometry (IMS) databases.

### 2.12. Statistical Analysis

All analyses were performed using three independent biological replicates, and the results are expressed as mean ± SD (*n* = 3). Individuals within each experimental group were randomly assigned to three biological replicates without additional selection or exclusion criteria. Each replicate consisted of 10 mussels, two of which were used for color, texture, and centrifugal loss measurements, while the remaining eight were pooled and homogenized for chemical analyses. Subsamples were taken from each pooled homogenate for the respective assays, and each biological replicate was treated as one statistical unit. Sex (male and female) and storage time (0, 90, and 150 days) were considered the two main experimental factors.

One-way analysis of variance followed by Duncan’s multiple range test was conducted using SPSS Statistics 26.0 (IBM Corp., Armonk, NY, USA), Graphs were generated using GraphPad Prism 10 (GraphPad Software, San Diego, CA, USA). Principal component analysis (PCA) and partial least squares-discriminant analysis (PLS-DA) were performed using MetaboAnalyst 6.0 (https://www.metaboanalyst.ca; accessed on 15 August 2026). Pearson correlation analysis non-volatile flavor compounds was conducted among was conducted using Origin Pro 2026 (OriginLab Corporation, Northampton, MA, USA) and visualized as correlation heatmaps. The heatmaps and correlation analyses between different classes of flavor-related compounds—including fatty acids, VOCs, amino acids and nucleotides—were generated using the ChiPlot online platform (https://www.chiplot.online/; accessed on 15 August 2026). Statistical significance was considered at *p* < 0.05.

## 3. Results and Discussion

### 3.1. Centrifugal Loss Analysis

**Figure 1:**
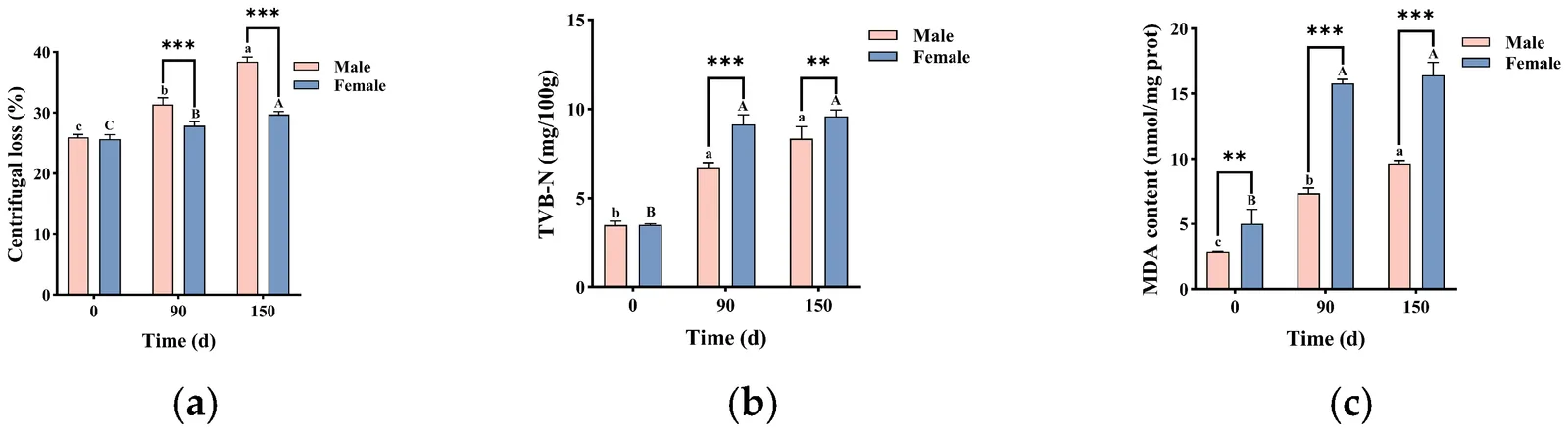

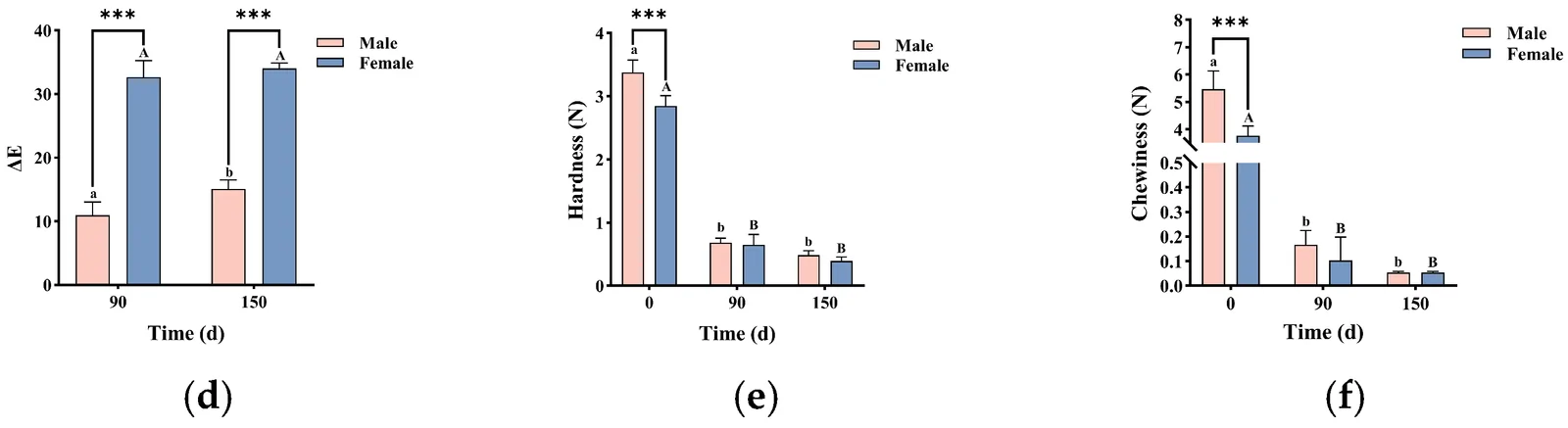
Changes in physicochemical quality attributes of male and female mussels during frozen storage. (**a**) Centrifugal loss. (**b**) TVB-N content. (**c**) MDA content. (**d**) ΔE. (**e**) Hardness. (**f**) Chewiness. Note: In (**a**–**f**), ** represents *p* < 0.01, *** represents *p* < 0.001. Different superscript lowercase letters (a, b, c) denote significant differences among storage times within males (*p* < 0.05), while different superscript uppercase letters (A, B, C) denote significant differences among storage times within females (*p* < 0.05); d: days.

Centrifugal loss reflects the water-holding capacity of muscle tissue and the ability of myofibrillar proteins to retain water. It is therefore closely related to the processing suitability and commercial quality of frozen products [15,30]. As shown in Figure 1a, centrifugal loss increased significantly in both female and male mussels with prolonged frozen storage (*p* < 0.05). No significant difference was observed between sexes in fresh samples; however, after freezing, the centrifugal loss was significantly higher in males than in females (*p* < 0.05) (Table S1). After 150 days of storage, the centrifugal loss increased to approximately 1.5 times the fresh level in males, compared with approximately 1.2 times in females, indicating a greater decline in water-holding capacity in male mussels during frozen storage. Previous studies have demonstrated that protein oxidation can alter the structural and functional properties of muscle proteins, thereby affecting texture, water-holding capacity, digestibility, and juiciness [14,31,32]. Moreover, sex-related differences in biochemical composition, lipid reserves, and muscle quality characteristics of aquatic animals may influence the extent of protein oxidation, water migration, and tissue fluid loss during frozen storage [33]. Taken together, the observed sex-associated differences in water-holding capacity may reflect differences in the physiological and biochemical responses of female and male mussels to frozen storage; however, the underlying mechanisms require further investigation.

### 3.2. Total Volatile Basic Nitrogen (TVB-N) Analysis

TVB-N is widely used as an indicator of freshness and the deterioration of nitrogenous compounds in aquatic products, reflecting the accumulation of volatile basic nitrogenous substances generated from the degradation of proteins, amines, and other non-protein nitrogenous compounds [34]. As shown in Figure 1b, no significant difference in TVB-N content was observed between female and male mussels in the fresh samples (Table S1). After 90 days of frozen storage, however, TVB-N was significantly higher in females than in males (9.14 ± 0.54 vs. 6.76 ± 0.24 mg/100 g, *p* < 0.05). Thereafter, the rate of TVB-N accumulation slowed in both groups. At the end of storage, TVB-N increased to approximately 2.4 times and 2.7 times the initial levels in males and females, respectively, but remained below the maximum limit of 15 mg/100 g specified for frozen shellfish in the Chinese National Food Safety Standard GB 2733-2015 [35]. This trend was consistent with the findings of Gao et al. [6]. Low-temperature storage can reduce endogenous enzyme activity and suppress microbial growth, thereby slowing the degradation of proteins and non-protein nitrogenous compounds and delaying TVB-N accumulation [36]. Previous studies have also shown that lipid and protein oxidation during frozen storage are interconnected through free-radical-mediated reactions and may mutually influence their progression [32,37]. Female mussels have been reported to contain higher lipid levels than males [24], and mussels are generally rich in polyunsaturated fatty acids (PUFAs). Previous research on *M. coruscus* demonstrated sex-related differences in lipid storage, with ovarian crude fat content reported to be higher than that of the testes [21]. Accordingly, such differences in lipid reserves, together with the higher MDA levels observed in females in the present study, may partly contribute to their greater susceptibility to lipid oxidation during frozen storage. However, further studies are needed to confirm this relationship.

### 3.3. MDA Analysis

MDA is a secondary product of lipid oxidation and is commonly used as an indicator of lipid peroxidation in aquatic products [38]. As shown in Figure 1c, MDA levels in males increased significantly from 0 to 90 days, with a further significant increase at 150 days, whereas in females, MDA levels increased significantly from 0 to 90 days but showed no significant difference between 90 and 150 days. In fresh samples, the MDA level in females was approximately 1.7 times that in males (*p* < 0.01). After 90 and 150 days of frozen storage, MDA levels remained significantly higher in females than in males (*p* < 0.001). At 150 days, MDA levels in both sexes were approximately 3.3 times their respective initial levels, while the level in females remained approximately 1.7 times that in males. These similar relative increases suggest a comparable progression of lipid oxidation between sexes, whereas the consistently higher absolute MDA level in females may primarily reflect differences in the amount of available lipid substrates. The fatty acid composition of endogenous lipids is an important determinant of the susceptibility of food lipids to peroxidation, with polyunsaturated fatty acids being particularly susceptible to the initiation and propagation of lipid oxidation [39,40]. Mussels are rich in polyunsaturated fatty acids, and disruption of muscle cell integrity during frozen storage may facilitate the release and interaction of pro-oxidative factors, including free radicals, metal ions, and oxidized lipids, thereby accelerating lipid oxidation and promoting MDA accumulation [30].

### 3.4. Color and Texture Analysis

Color and texture are important quality attributes of aquatic products and strongly influence consumer purchase decisions [8]. The total color difference was calculated from the L_1_, a_1_, and b_1_ values measured using a colorimeter. For each sex, the corresponding fresh sample at 0 d was used as the reference to evaluate the overall color deviation induced by frozen storage, with a higher ΔE indicating a greater departure from the initial color state [41]. As shown in Figure 1d, female mussels exhibited a pronounced color change within the first 90 days of frozen storage, with a ΔE of 32.66 ± 2.59, whereas no significant difference was observed between 90 and 150 days. In contrast, ΔE in male mussels increased progressively from 10.97 ± 2.07 at 90 days to 15.07 ± 1.40 at 150 days (Table S1). The higher ΔE observed in females may be associated with their greater extent of lipid oxidation. Aldehydes and other reactive carbonyl compounds generated during lipid oxidation can react with amino groups in proteins through condensation and polymerization reactions, contributing to the formation of yellow or brown pigments and thereby altering the overall color of aquatic products [42,43]. This interpretation is consistent with the higher MDA levels observed in female mussels during the later stages of frozen storage. In addition, protein denaturation and changes in water distribution during freezing may also affect tissue lightness and overall color appearance [44].

Texture is an important indicator of muscle quality and is closely associated with ice crystal size, water loss, and tissue integrity, while being susceptible to protein denaturation and structural changes in muscle during frozen storage [15,30]. As shown in Figure 1e,f, hardness and chewiness decreased significantly in both female and male mussels after frozen storage (*p* < 0.05). In fresh samples, males exhibited significantly higher hardness and chewiness than females (*p* < 0.001), indicating a firmer tissue structure in male mussels under the fresh condition (Table S1). After 90 days of frozen storage, both texture parameters decreased markedly in both sexes, with no significant differences between 90 and 150 days, suggesting that most of the texture deterioration occurred during the first 90 days of storage. These changes were consistent with the increase in centrifugal loss. Water plays an important role in maintaining muscle structure, and freeze-induced water loss can adversely affect muscle texture and palatability [45]. In addition, frozen storage can cause disruption of muscle fibers, breakdown of connective tissue, and shrinkage of muscle bundles, thereby compromising tissue integrity [3,6]. These structural alterations, together with progressive water loss, may account for the pronounced decreases in hardness and chewiness observed in both female and male mussels.

### 3.5. Free Amino Acids (FAAs) Analysis

**Figure 2:**
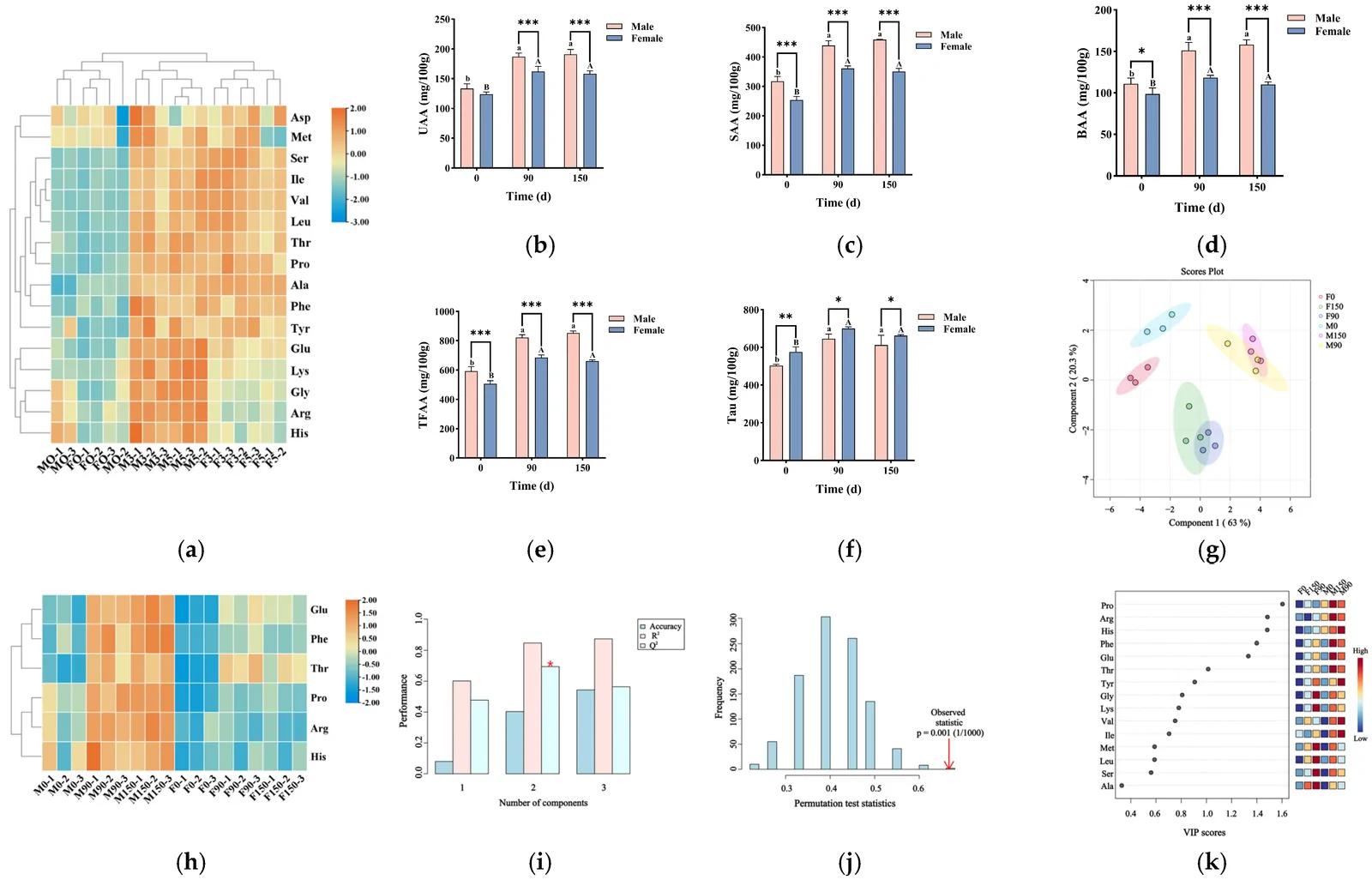
Changes in free amino acid profiles and multivariate analysis of male and female mussels during frozen storage. (**a**) FAA heatmap. (**b**) UAAs. (**c**) SAAs. (**d**) BAAs. (**e**) TFAAs. (**f**) Tau. (**g**) PLS-DA score plot. (**h**) Hierarchical clustering heatmap of the selected differential FAAs. (**i**) Cross-validation results of the PLS-DA model, an asterisks (*) indicates statistical significance (*p* < 0.05). (**j**) Permutation test of the PLS-DA model (*n* = 1000). (**k**) VIP score plot highlighting key discriminant compounds. Note: In (**b**–**f**), * indicate the differences between males and females at the same storage time. * represents *p* < 0.05, ** represents *p* < 0.01, *** represents *p* < 0.001. Different superscript lowercase letters (a, b) denote significant differences among storage times within males (*p* < 0.05), while different superscript uppercase letters (A, B) denote significant differences among storage times within females (*p* < 0.05); d: days.

Free amino acids are important contributors to taste and can also serve as precursors for the formation of aroma compounds [46]. A total of 16 FAAs were detected in the mussel samples, and their taste characteristics varied according to their chemical structures (Table S2). Figure 2a presents the hierarchical clustering heatmap of FAA profiles in mussel tissues. In the fresh samples, the overall FAA compositions of female and male mussels were relatively similar. As frozen storage progressed, FAA profiles gradually diverged between the two sexes. Based on their taste characteristics, FAAs were classified into three groups: UAAs, sweet amino acids (SAAs), and bitter amino acids (BAAs). As shown in Figure 2b–e, the contents of UAAs, SAAs, BAAs, and total free amino acids (TFAAs) increased in both sexes, while the rates of increase tended to slow during the later stage. A similar pattern has been reported in frozen *Parapenaeus longirostris*, in which most FAAs accumulated during the first 6–8 months of storage and subsequently declined [47]. Freeze-thaw treatment has likewise been reported to increase FAA levels in cuttlefish [48]. During freezing, disruption of lysosomal membranes may promote the release of endogenous hydrolytic enzymes, thereby facilitating the degradation of proteins and peptides and contributing to FAA accumulation [49]. Compared with females, male mussels generally contained higher levels of UAAs, SAAs, BAAs, and TFAAs throughout storage, and these differences became more pronounced after freezing (*p* < 0.05). The consistently higher FAA levels in males may be related to their greater protein reserves, which could provide more substrate for protein degradation and amino acid release during prolonged frozen storage.

The behavior of taurine (Tau) differed from that of the other FAA groups (Figure 2f). Tau levels were significantly higher in females than in males at all storage stages (*p* < 0.05). In both sexes, Tau increased after 90 days of frozen storage and then decreased slightly or tended to stabilize by 150 days. Tau is an abundant free amino acid-like compound in shellfish and is involved in osmotic regulation, cellular protection, and taste characteristics [50]. The higher Tau levels observed in females may therefore reflect sex-related differences in FAA composition and osmoregulatory metabolism. Ice crystal formation and changes in the unfrozen phase can damage cellular and myofibrillar structures, impair membrane integrity, and promote protein denaturation and degradation, thereby facilitating FAA accumulation [14,47]. These structural alterations are consistent with the reduced water-holding capacity, increased tissue fluid loss, and deterioration in color and texture observed during frozen storage.

Partial least-squares discriminant analysis (PLS-DA) was performed to evaluate differences in FAA profiles among the mussel groups. Model performance was assessed using 5-fold cross-validation and permutation testing. Based on the highest *Q*^2^ value, the optimal model contained three components, with an accuracy of 0.543, *R*^2^ of 0.872, and *Q*^2^ of 0.564. Permutation testing (*n* = 1000) yielded *p* = 0.001, indicating that the observed group discrimination was unlikely to have arisen by chance (Figure 2i,j). The first two latent variables explained 63.0% and 20.3% of the variation, respectively, accounting for 83.3% in total, and clearly separated the six groups in the score plot (Figure 2g). Biological replicates clustered closely within each group, indicating good reproducibility. The six groups were clearly separated in the PLS-DA score plot, suggesting that both storage duration and sex influenced FAA profiles. F90 and F150 clustered closely, as did M90 and M150, indicating that most changes occurred within the first 90 days of frozen storage. Meanwhile, the increasing separation between female and male groups after freezing suggested that storage further accentuated sex-related differences in FAA composition. Previous metabolomic studies on mussels have shown that FAA distribution exhibits clear tissue- and sex-dependent patterns [22].

Variable importance in projection (VIP) analysis (Figure 2k) identified six amino acids with VIP values > 1, including Glu, Phe, Thr, Pro, Arg and His. The corresponding clustering heatmap (Figure 2h) further revealed distinct sex-related distribution patterns among these key discriminative amino acids. In females, frozen storage was mainly characterized by increases in Gly and Thr, whereas Pro, Phe, His, and Arg showed comparatively smaller changes. In contrast, several BAAs, particularly Phe, His, and Arg, accumulated progressively in males with prolonged storage. During storage, FAA may undergo further conversion or depletion through pathways such as deamination and decarboxylation, which could contribute to the differential temporal patterns observed among individual amino acids [51].

### 3.6. 5′-Nucleotide Analysis

**Figure 3:**
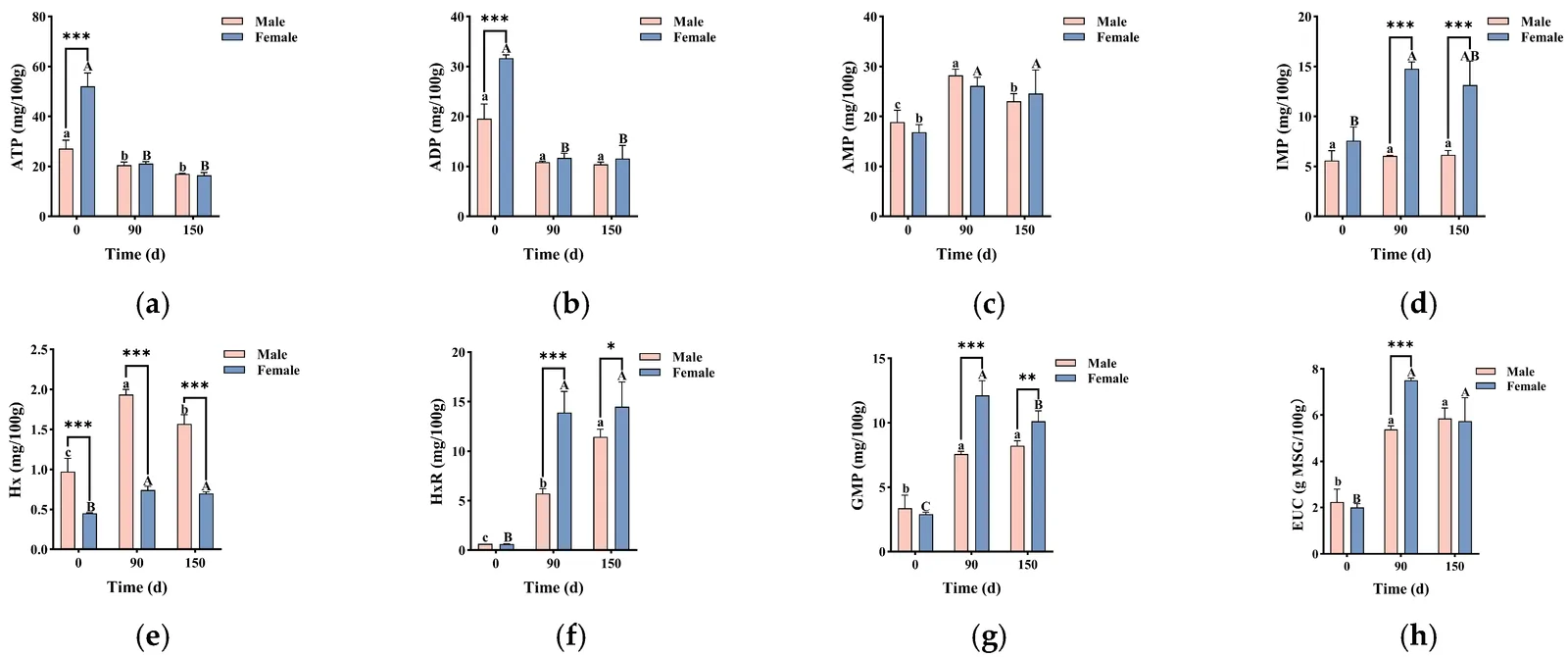
Changes in ATP-related compounds and EUC in male and female mussels during frozen storage. (**a**) ATP. (**b**) ADP. (**c**) AMP. (**d**) IMP. (**e**) Hypoxanthine (Hx). (**f**) Inosine (HxR). (**g**) GMP. (**h**) EUC. Note: In (**a**–**h**), * indicate the differences between males and females at the same storage time. * represents *p* < 0.05, ** represents *p* < 0.01, *** represents *p* < 0.001. Different superscript lowercase letters (a, b, c) denote significant differences among storage times within males (*p* < 0.05), while different superscript uppercase letters (A, B, C) denote significant differences among storage times within females (*p* < 0.05); d: days.

Nucleotides are important nitrogen-containing compounds in aquatic products and contribute substantially to their characteristic taste. A total of eight nucleotides and related compounds were detected in mussel tissues (Table S1). ATP and adenosine diphosphate (ADP) levels decreased significantly in both female and male mussels during storage (Figure 3a,b), with no significant differences between 90 and 150 days, suggesting that most of their degradation occurred during the first 90 days of frozen storage. Wang et al. similarly reported rapid ATP degradation during the early stage of frozen storage, whereas ADP decreased progressively with storage time [52]. Ice crystal formation during freezing can disrupt cellular membranes and tissue structure, thereby increasing contact between endogenous enzymes and their substrates, while residual enzymatic activity may further promote the conversion of ATP to ADP and AMP [53].

Consistent with ATP and ADP degradation, AMP increased significantly after 90 days of frozen storage, with no sex-related differences. In contrast, IMP and GMP showed clear sex-dependent patterns, with significantly higher levels in females than in males at 90 and 150 days, particularly for IMP (*p* < 0.001). AMP, IMP, and GMP are important taste-active nucleotides that contribute to umami perception [4]. Their accumulation, particularly the higher IMP and GMP levels in females, may act synergistically with monosodium glutamate and umami-tasting amino acids to strengthen overall umami perception [9]. With storage, inosine (HxR) levels increased significantly in both mussels, with higher levels observed in females at 90 and 150 days. In contrast, hypoxanthine (Hx) remained significantly higher in males throughout storage (*p* < 0.001). ATP can be sequentially degraded by endogenous enzymes to HxR and Hx, and excessive accumulation of these degradation products may contribute to bitterness and other undesirable taste attributes in aquatic products [46,54].

EUC, which expresses the combined umami contribution of these compounds relative to monosodium glutamate, has been widely used to evaluate the potential umami intensity of foods [46]. The EUC increased markedly in both sexes after frozen storage (Figure 3h). In females, the EUC reached a maximum of 7.51 ± 0.09 g MSG/100 g at 90 days, significantly exceeding that of males at the same time point (5.38 ± 0.15 g MSG/100 g) (Table S1). This increase was associated with the concurrent accumulation of taste-active compounds, including Glu, Asp, IMP, and GMP. Overall, female mussels exhibited higher IMP, GMP, and EUC at 90 days, whereas males accumulated more Hx, suggesting sex-dependent patterns in ATP-related metabolite conversion and responses to frozen storage stress [55,56].

### 3.7. Fatty Acids Analysis

**Figure 4:**
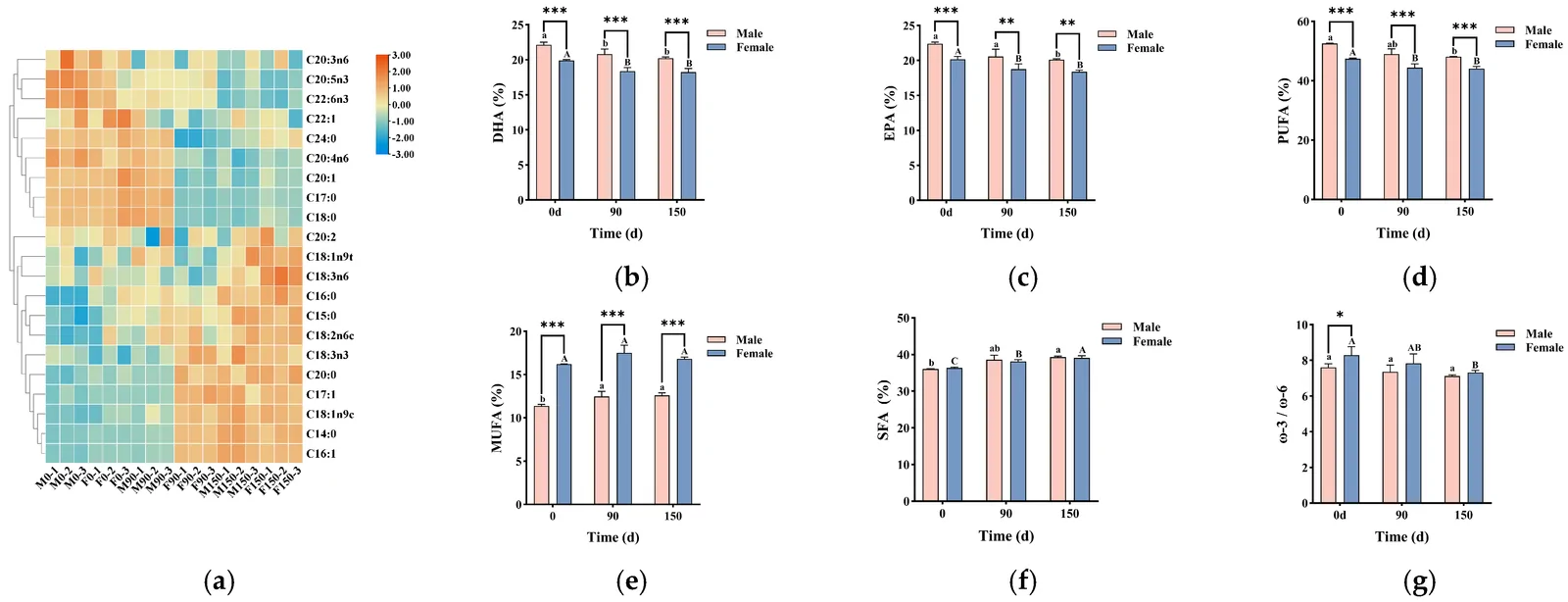
Changes in fatty acid compositions of male and female mussels during frozen storage. (**a**) FAMEs heatmap. (**b**) DHA. (**c**) EPA. (**d**) PUFAs. (**e**) MUFAs. (**f**) SFAs. (**g**) ω-3/ω-6 fatty acid ratio. Note: In (**b**–**g**), * indicate the differences between males and females at the same storage time. * represents *p* < 0.05, ** represents *p* < 0.01, *** represents *p* < 0.001. Different superscript lowercase letters (a, b) denote significant differences among storage times within males (*p* < 0.05), while different superscript uppercase letters (A, B, C) denote significant differences among storage times within females (*p* < 0.05); d: days.

Owing to their high degree of unsaturation, ω-3 polyunsaturated fatty acids (ω-3 PUFAs) are particularly susceptible to oxidative degradation. Although various biochemical and microbial processes may occur simultaneously during storage, lipid oxidation is considered one of the major factors contributing to quality deterioration and shelf-life reduction of marine foods [39,49]. As shown in Figure 4a, a total of 21 fatty acids were identified in the mussel samples, including 6 monounsaturated fatty acids (MUFAs), 8 PUFAs, and 7 saturated fatty acids (SFAs). Unsaturated fatty acids accounted for approximately 62% of the total fatty acids, whereas SFAs represented approximately 36% (Table S3). Among the individual fatty acids, C16:0 was the most abundant, accounting for approximately 25% of the total, followed by DHA and EPA, each contributing approximately 20%. This fatty acid profile is consistent with previous findings for mussels [4,26]. M0 and F0 exhibited similar fatty acid profiles (Figure 4a), indicating limited sex-related differences in fresh mussels. After freezing, females and males showed distinct temporal patterns: F90 clustered with F150 and M150, whereas M90 remained closer to the fresh samples. This suggests that fatty acid composition changed earlier in females and then became relatively stable, while changes in males occurred more gradually over storage.

As shown in Figure 4b–g, frozen storage altered the relative fatty acid composition of mussels. DHA, EPA, and total PUFAs generally decreased, with earlier changes in females and more gradual decreases in males. Males consistently exhibited higher proportions of DHA, EPA, and total PUFAs, whereas MUFAs were higher in females. In contrast, MUFAs and SFAs generally increased during storage. The ω-3/ω-6 ratio declined progressively in females but remained relatively stable in males, indicating distinct sex-dependent changes in fatty acid profiles during frozen storage. These sex-related differences may be associated with variations in lipid reserves, lipid distribution, and fatty acid composition between female and male mussels [26]. A recent direct comparison of female and male *Mytilus edulis* likewise revealed sex-dependent differences in lipid and fatty acid composition, with males exhibiting a higher combined proportion of EPA and DHA than females [19], further supporting the influence of sex on mussel lipid profiles. Freezing slows but does not completely prevent lipid oxidation in aquatic products. During prolonged frozen storage, triglycerides and phospholipids may undergo hydrolysis and oxidation, accompanied by changes in free fatty acids and the accumulation of lipid oxidation products such as MDA [18,49]. The earlier shift in fatty acid composition observed in females was consistent with their higher MDA levels, suggesting a greater accumulation of lipid oxidation products in female mussels during long-term frozen storage. This difference may be related, at least in part, to their higher lipid reserves and consequently greater availability of lipid substrates for oxidation.

### 3.8. Volatile Compounds

#### 3.8.1. Odor Profile Analysis

**Figure 5:**
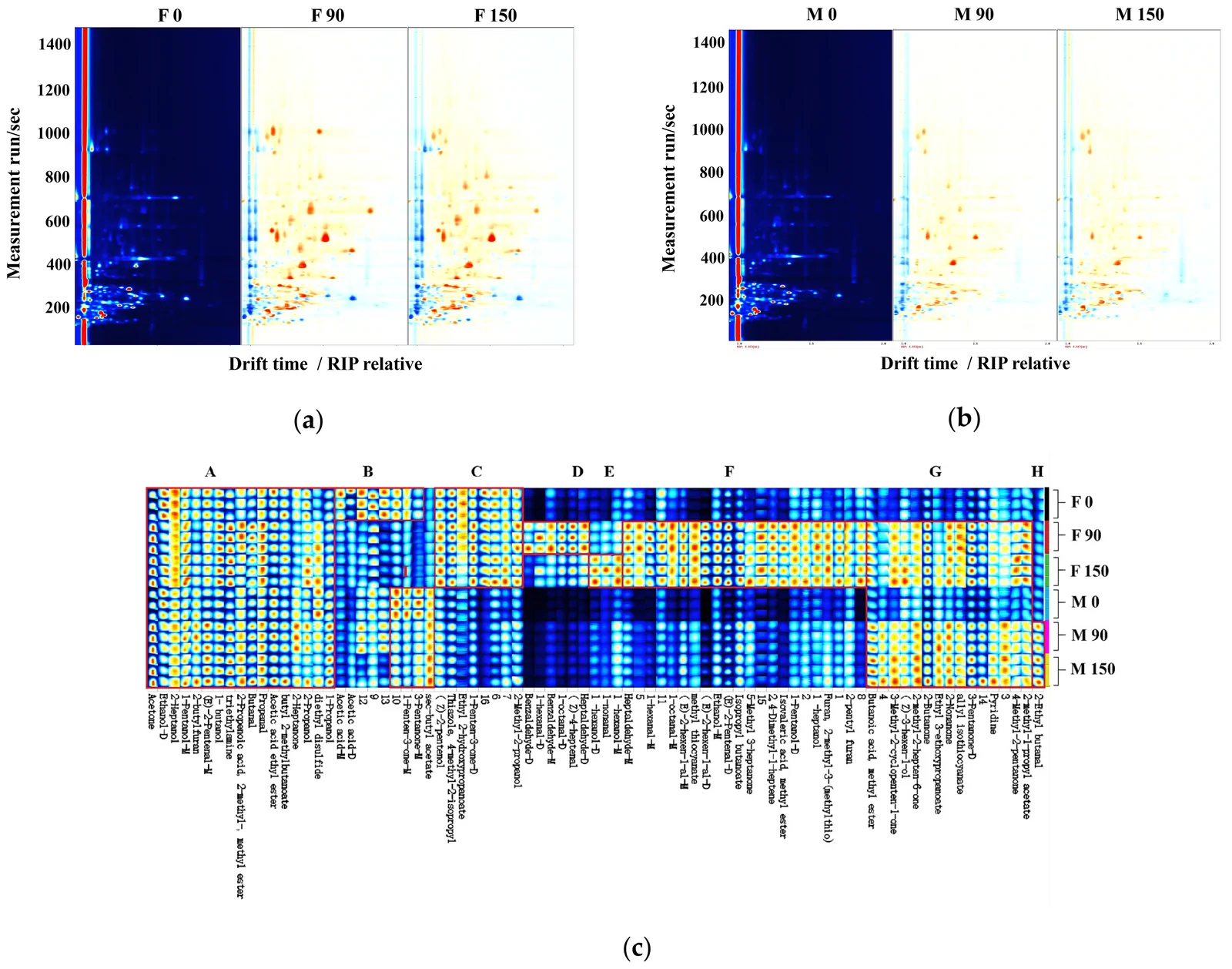
Difference plots of VOCs in male and female *Mytilus coruscus* during frozen storage based on GC-IMS. (**a**) Female groups with F0 as the reference. (**b**) Male groups with M0 as the reference. (**c**) Gallery plot generated from GC-IMS data. Note: In the gallery plot, each row represents a sample, whereas each column represents a VOC. The color represents the relative signal intensity of each VOC, ranging from white (low intensity) to red (high intensity); a deeper red color indicates a higher relative abundance.

To further compare changes in VOC profiles before and after frozen storage, fresh male (M0) and female (F0) samples were used as the respective references, and difference plots were generated using the subtraction function of the GC-IMS software (Figure 5a,b). After applying the “Verify” function, the background was normalized to white. Red regions indicate VOC signals with higher relative intensities in the frozen samples than in the corresponding fresh reference, whereas blue regions indicate lower relative intensities; greater color intensity represents a larger difference. The VOC fingerprints of both female and male mussels changed markedly after 90 days of frozen storage, whereas comparatively smaller changes were observed between 90 and 150 days. A similar pattern was reported by Zhan et al. in frozen drunken shrimp [57]. Distinct regions of increased and decreased signal intensity were observed at different storage stages, indicating that frozen storage involved the concurrent formation, accumulation, transformation, and loss of multiple volatile compounds. In addition, the temporal patterns of VOC changes differed between females and males, suggesting a sex-dependent response of the volatile profile to frozen storage [18].

Variations in VOC profiles among mussels at different storage stages were further visualized using the GC-IMS fingerprint plot (Figure 5c). A total of 81 volatile signals were detected, comprising 17 aldehydes, 13 alcohols, 12 ketones, 10 esters, 3 furans, 2 acids, 6 sulfur- or nitrogen-containing compounds, 1 hydrocarbon, and 16 other compounds (Table S4). The volatiles located in region A (Figure 5c, region A) were consistently detected in both fresh and frozen samples and included alcohols, aldehydes, ketones, esters, sulfur-containing compounds, and amines, representing a common baseline volatile profile of the mussels. As storage progressed, the distribution patterns of VOC gradually diverged among the groups. Some characteristic volatiles abundant in fresh samples decreased, whereas other compounds potentially associated with lipid oxidation, protein degradation, and the transformation of nitrogen-containing constituents accumulated during frozen storage. These changes may arise from the combined contributions of enzymatic and non-enzymatic reactions [17].

Distinct sex-related differences in volatile profiles were already evident in fresh *M. coruscus* (Figure 5c, regions B, C, and I). Compared with M0, F0 exhibited higher relative signal intensities of acetic acid, (Z)-2-pentenol, ethyl 2-hydroxypropanoate, 2-methyl-2-propanol, and Thiazole, 4-methyl-2-isopropyl, with acetic acid showing strong signals in both its monomeric and dimeric forms. In contrast, 1-penten-3-one, 3-pentanone, and sec-butyl acetate showed higher relative signal intensities in M0. Similar sex-related differences in VOC profiles have also been reported in other bivalves. Fu et al. found higher acetic acid levels in female Pacific oysters, whereas 3-pentanone was more abundant in males [58]. Zhao et al. likewise demonstrated that sex influenced VOC composition and formation patterns in mussels [18].

After 90 days of frozen storage, the VOC profiles of female and male mussels diverged further. F90 was characterized by increased aldehyde signals in region D, particularly those of benzaldehyde, hexanal, octanal, (Z)-4-heptenal, and heptanal. Aliphatic aldehydes such as hexanal, heptanal, and octanal are associated with green, fatty, and fruity odor notes, whereas (Z)-4-heptenal has been closely linked to marine lipid oxidation and fishy off-odors [58]. In addition, F90 and F150 maintained relatively high signals of heptanal, hexanal, octanal, and (E)-2-hexenal in region F, together with several alcohols, furans, and sulfur-containing compounds. Because aldehydes generally have low odor thresholds, even relatively low abundances may contribute substantially to the overall aroma of shellfish [4]. The enrichment of these aldehydes, together with the higher MDA levels and earlier changes in fatty acid composition observed in females, suggests that lipid oxidation may represent an important pathway associated with VOC remodeling in female mussels during frozen storage [59].

In comparison, fewer VOCs were specifically enriched in M90, with 2-ethylbutanal being one of the more prominent compounds, while several ketones and esters maintained relatively high signal intensities across the male storage groups. When storage was extended from 90 to 150 days, the signals of several aldehydes in females, including benzaldehyde, hexanal, octanal, (Z)-4-heptenal, and heptanal, decreased markedly, suggesting that these compounds may have accumulated during the intermediate stage of frozen storage and subsequently undergone further transformation or loss. In contrast, the overall VOC profile of male mussels changed comparatively little during this period. Overall, females exhibited more pronounced aldehyde accumulation followed by a subsequent decline during prolonged frozen storage, whereas the male VOC profile remained relatively stable, indicating distinct sex-dependent trajectories of volatile flavor evolution in *M. coruscus*. Similar sex-dependent differences in VOC composition have been reported in other bivalves [58].

#### 3.8.2. PCA and PLS-DA of Odor Profile Analysis

**Figure 6:**
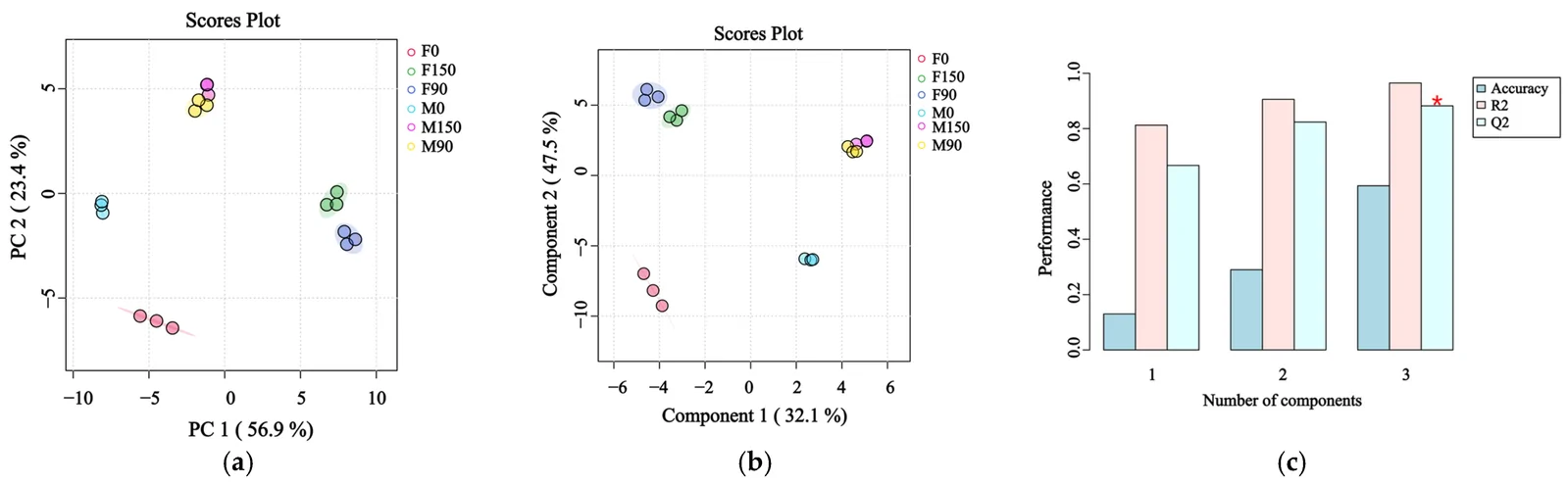

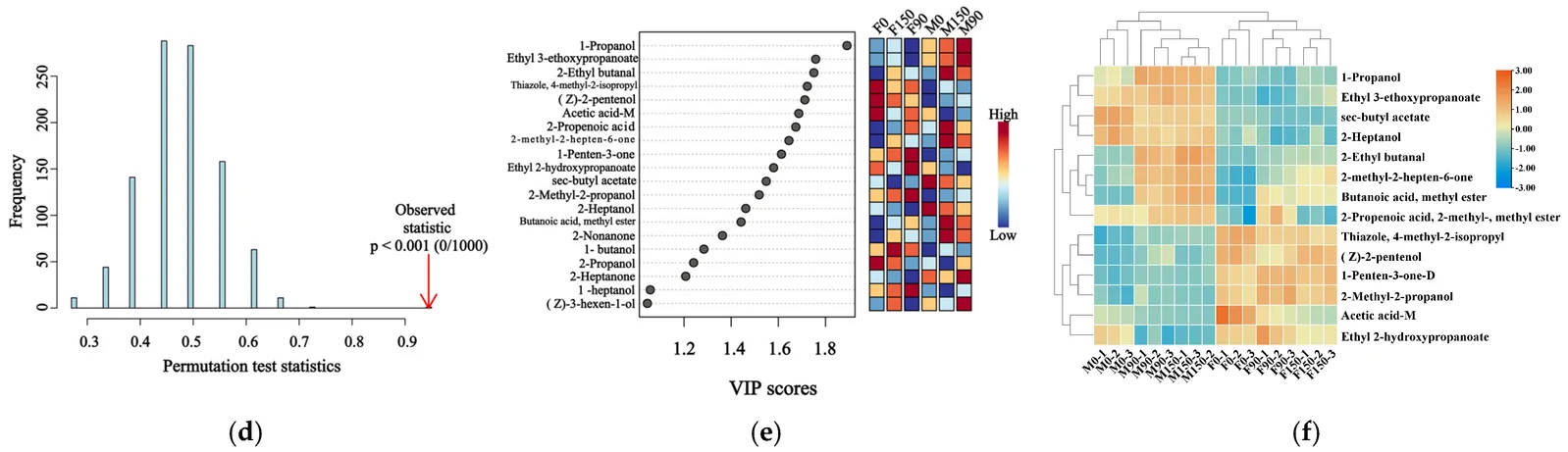
(**a**) PCA based on GC-IMS volatile profiles. (**b**) PLS-DA score plot of VOC profiles. (**c**) Cross-validation (CV) results indicate model prediction accuracy. Q2 values (predictive ability) with asterisks (*) denote statistical significance (*p* < 0.05), indicating that the model outperforms random chance; (**d**) Permutation test statistics (*n* = 1000) validating model significance (*p* < 0.001). (**e**) VIP scores plot highlighting key discriminant compounds. (**f**) Hierarchical clustering heatmap of the selected differential VOCs.

As shown in Figure 6a, the first two principal components of the PCA model, PC1 and PC2, explained 56.9% and 23.4% of the total variance, respectively, accounting for 80.3% cumulatively. The three biological replicates within each group clustered closely, indicating good within-group reproducibility. M0 and F0 were clearly separated in the score plot, demonstrating that sex-related differences in volatile profiles were already present in fresh *M. coruscus* before frozen storage. After 90 days of storage, both F90 and M90 shifted markedly away from their corresponding fresh groups, indicating substantial remodeling of the overall VOC profiles during the early stage of frozen storage. Notably, F90 clustered close to F150, while M90 and M150 also showed substantial overlap, suggesting that further changes in the overall VOC composition between 90 and 150 days were relatively limited compared with those occurring during the first 90 days. In addition, frozen female samples were mainly distributed along the positive direction of PC1, whereas frozen male samples were primarily located in the positive region of PC2, further suggesting distinct sex-dependent trajectories of VOC changes during frozen storage.

PLS-DA further improved discrimination among the six groups (Figure 6b), with the first two latent variables explaining 79.6% of the total variation. The optimal three-component model showed good performance, with an accuracy of 0.883, R^2^ of 0.986, and Q^2^ of 0.922 based on 5-fold cross-validation (Figure 6c). Permutation testing (*n* = 1000) yielded *p* < 0.001 (Figure 6d), indicating that the observed group discrimination was unlikely to result from random variation. Clear separation between F0 and M0 further confirmed sex-related differences in VOC composition. The greater distances between fresh and frozen groups than between the 90 and 150 days groups suggested that most VOC remodeling occurred within the first 90 days of storage. M90 and M150 clustered particularly closely, whereas F90 and F150 showed slightly greater separation, indicating comparatively limited later-stage changes in males and continued modest adjustment of the female VOC profile. This may be because the higher lipid content of female mussels may provide a larger pool of oxidation-prone lipid substrates, which could sustain the initiation and propagation of lipid oxidation during prolonged frozen storage [19,39].

To further identify the volatile compounds contributing most strongly to group discrimination, VOCs were screened according to the VIP scores derived from the PLS-DA model [18]. Because VIP values > 1 are generally considered indicative of a relatively high contribution to model discrimination, a more stringent threshold of VIP > 1.4 was applied in the present study. A total of 14 key discriminative VOCs were identified (Figure 6e), comprising five esters, four alcohols, two ketones, one aldehyde, one acid, and one nitrogen-sulfur containing heterocyclic compound. Esters and alcohols represented the major classes, while carbonyl compounds also contributed substantially to group discrimination. Ketones in aquatic products may originate from the oxidation and degradation of polyunsaturated fatty acids, amino acid degradation, microbial metabolism, and the oxidation of secondary alcohols [4,58]. Aldehydes are mainly generated through oxidative degradation of unsaturated fatty acids, whereas esters are commonly formed through esterification reactions between alcohols and carboxylic acids [60].

Hierarchical clustering of the 14 key discriminative VOCs (Figure 6f) revealed two major clusters with broadly opposite sex-related distribution patterns. The sample dendrogram also largely separated female and male mussels, further indicating that sex was an important source of variation in the volatile profiles. Similar sex-dependent differences in VOC composition have been reported in Pacific oysters and mussels [18,58]. The first VOC cluster, including 1-propanol, ethyl 3-ethoxypropanoate, sec-butyl acetate, 2-heptanol, 2-ethylbutanal, 2-methyl-2-hepten-6-one, methyl butanoate, and methyl 2-methylpropenoate, generally exhibited higher standardized signal intensities in male samples. These observations are consistent with previous results [58]. In contrast, 4-methyl-2-isopropylthiazole, (Z)-2-pentenol, 1-penten-3-one-D, 2-methyl-2-propanol, acetic acid-M, and ethyl 2-hydroxypropanoate were predominantly associated with female samples. These opposing patterns suggest that sex affected not only the overall VOC profile but also the relative distribution of individual key discriminative compounds during frozen storage.

#### 3.8.3. Interactions Among Fatty Acids, Volatile Compounds and Non-Volatile Flavor Substances

**Figure 7:**
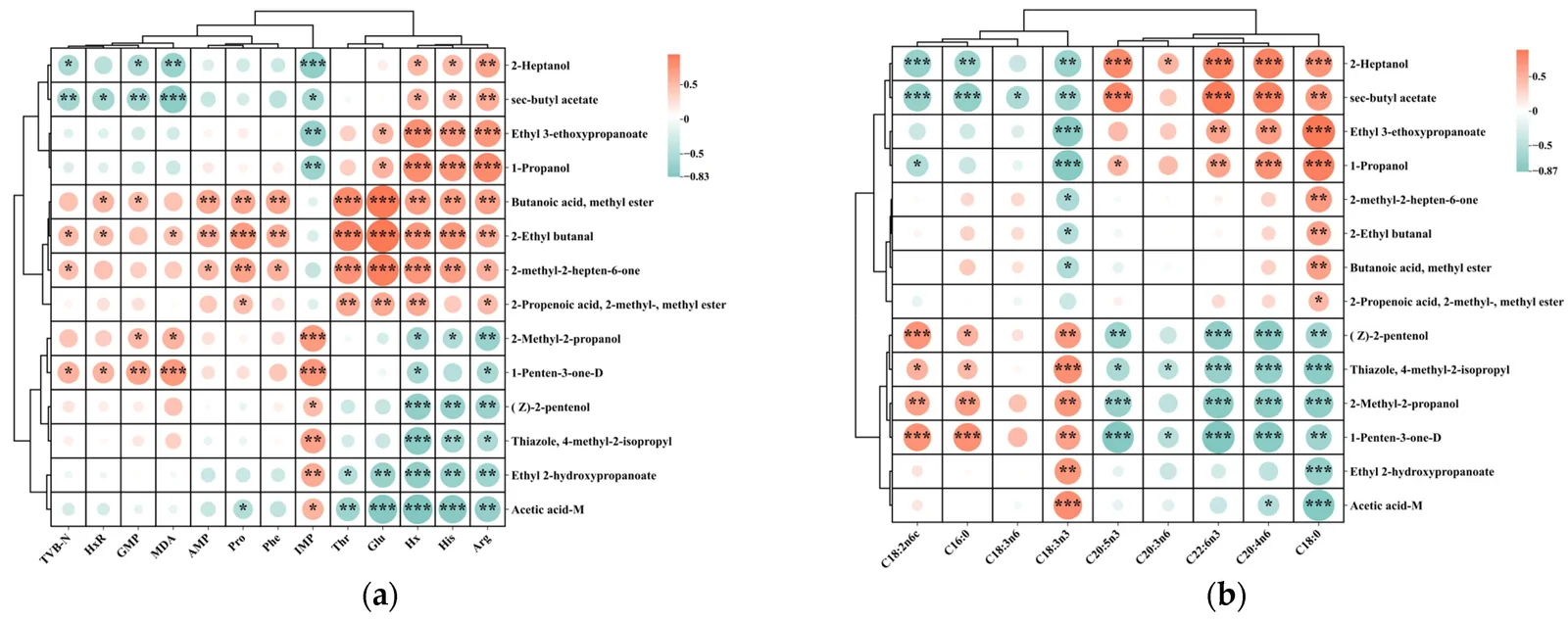
(**a**) Pearson correlation heatmap between non-volatile flavor compounds (free amino acids, TVB-N, MDA and 5′-nucleotides) and differential volatile compounds. (**b**) Pearson correlation heatmap between unsaturated fatty acids and differential volatile compounds. Note: In the correlation heatmap, red indicates a positive correlation, whereas blue indicates a negative correlation; deeper colors represent stronger correlations. Asterisks indicate statistical significance (* represents *p* < 0.05, ** represents *p* < 0.01, *** represents *p* < 0.001).

Pearson correlation analyses were performed using data from all 18 independent biological replicates (6 experimental groups × 3 biological replicates). Correlation analysis revealed broadly consistent association patterns between the key discriminative VOCs and taste-related metabolites, quality indices, and fatty acid composition (Figure 7a,b). 2-Heptanol, sec-butyl acetate, ethyl 3-ethoxypropanoate, and 1-propanol were generally positively correlated with Hx but negatively correlated with IMP and several taste-related amino acids; these VOCs were also positively associated with the long-chain PUFAs EPA, DHA, and ARA, but negatively associated with C18:3n3. In contrast, (Z)-2-pentenol, 4-methyl-2-isopropylthiazole, 2-methyl-2-propanol, and 1-penten-3-one-D exhibited broadly opposite patterns, showing positive associations with IMP, Gly, Glu, C18:3n3, and C18:2n6c, but negative associations with Hx, EPA, DHA, and ARA. Notably, 1-penten-3-one-D was also positively correlated with MDA, further suggesting an association between changes in this volatile signal and lipid oxidation. Similar relationships between lipid oxidation, long-chain fatty acid changes, and VOC formation have been reported in aquatic products [16]. Collectively, the complementary patterns observed in the two correlation analyses suggest that VOC remodeling during frozen storage occurred concurrently with nucleotide degradation, FAA changes, lipid oxidation, and shifts in fatty acid composition [12,16,60].

## 4. Conclusions

This study demonstrated that female and male *Mytilus coruscus* differed not only in their initial biochemical characteristics but also in their quality and flavor-related chemical responses during frozen storage. Males generally contained higher levels of free amino acids and exhibited a greater decline in water-holding capacity, whereas females showed greater susceptibility to lipid oxidation. The evolution of free amino acids also differed between sexes: females were characterized mainly by increases in Gly and Thr with relatively limited changes in Pro, Phe, His, and Arg, while several bitter amino acids, particularly Phe, His, and Arg, progressively accumulated in males. Sex-associated differences were also evident in ATP-related compounds, with Hx being more prominent in males, whereas other ATP-related metabolites were generally higher in females and changed markedly during storage. Meanwhile, frozen storage shifted the fatty acid profile toward a higher proportion of saturated fatty acids and a lower proportion of unsaturated fatty acids, with males generally retaining higher levels of unsaturated fatty acids.

Distinct sex-associated trajectories were further observed in VOC profiles. Females exhibited more pronounced changes in aldehyde-related signals, characterized by accumulation followed by a decline during prolonged storage, whereas the overall VOC profile of males remained comparatively stable. Key discriminative VOCs also showed contrasting associations with male and female samples. Correlation analysis further indicated that these VOC changes occurred concurrently with nucleotide degradation, FAA variation, lipid oxidation, and shifts in fatty acid composition, suggesting coordinated changes among taste-related metabolites, lipid-derived components, and volatile compounds during frozen storage. Overall, these findings demonstrate that sex represents an important biological source of variation in the frozen-storage quality of *M. coruscus* under frozen storage and should be considered in quality evaluation, raw-material classification, and the storage management of frozen mussels.

A major strength of this study is the integrated evaluation of multiple quality- and flavor-related indicators within the same experimental framework. Nevertheless, the study was limited by the use of a single storage temperature, three sampling points, relative rather than absolute fatty acid quantification, and the absence of sensory evaluation. Seasonal and reproductive-stage variation was not assessed, and the limited number of biological replicates precluded formal sex × storage time interaction analysis and extensive sex-stratified correlations. Future studies incorporating broader storage conditions, larger sample sizes, sensory evaluation, and seasonal and reproductive-stage comparisons will further clarify the mechanisms underlying sex-associated quality changes during frozen storage.

## Data Availability

The original contributions presented in the study are included in the article/Supplementary Materials, further inquiries can be directed to the corresponding authors.

## Supplementary Materials

The following supporting information can be downloaded at: https://www.mdpi.com/article/10.3390/foods15183351/s1, Table S1: Changes in quality attributes, ATP-related compounds, and equivalent umami concentration of female and male mussels during frozen storage, Table S2: Changes in free amino acids of female and male mussels during frozen storage (mg/100 g), Table S3: Changes in fatty acid composition of female and male mussels during frozen storage (% of total fatty acids), Table S4: Differences in volatile flavor compounds between male and female mussels.

## References

[1] Qian J., Deng F., Shumway S.E., Hu M., Wang Y. (2024). The thick-shell mussel *Mytilus coruscus*: Ecology, physiology, and aquaculture. Aquaculture.

[2] Bureau of Fisheries, Ministry of Agriculture and Rural Affairs, National Fisheries Technology Extension Center, China Society of Fisheries (2026). China Fishery Statistical Yearbook 2026.

[3] Strateva M., Stratev D., Zhelyazkov G. (2023). Freezing influence on the histological structure of Mediterranean mussel (*Mytilus galloprovincialis*). AIMS Agric. Food.

[4] Wu S., Ma H., Liu Y., Shi H., Xue C., Chen L., Li Z. (2025). Effects of cooling rate on the physiological metabolism and flavor of thick-shell mussel (*Mytilus coruscus*) during low-temperature semi-anhydrous living-preservation. Aquaculture.

[5] Du X., Wang B., Li H., Liu H., Shi S., Feng J., Pan N., Xia X. (2022). Research progress on quality deterioration mechanism and control technology of frozen muscle foods. Compr. Rev. Food Sci. Food Saf..

[6] Gao Y., Jiang H., Lv D., Benjakul S., Zhang B. (2021). Shelf-Life of Half-Shell Mussel (*Mytilus edulis*) as Affected by Pullulan, Acidic Electrolyzed Water, and Stable Chlorine Dioxide Combined Ice-Glazing during Frozen Storage. Foods.

[7] Liu Y., Tan Y., Luo Y., Li X., Hong H. (2024). Evidence of myofibrillar protein oxidation and degradation induced by exudates during the thawing process of bighead carp fillets. Food Chem..

[8] Jian W., Gao J., Xia X., Cao W., Lin H., Zheng H., Zhu G., Zheng Z. (2026). Magnetic field-assisted freezing mitigates quality deterioration in oyster meat (*Crassostrea hongkongensis*) by inducing the formation of uniform ice crystals and preserving water-holding capacity. Food Chem..

[9] Li L., Fu Z., Liu Y., Song Z., Yang X., Yu D., Wang Q., Chi H., Zheng J. (2025). A Comprehensive and Comparative Study on the Biochemical Composition and Non-Volatile Taste Compounds of Thirteen Shellfish Species. Foods.

[10] Zhang C., Shi R., Mi S., Chitrakar B., Liu W., Xu Z., Sang Y., Yu W., Wang X. (2024). Effect of different thermal processing methods on flavor characteristics of *Penaeus vannamei*. LWT.

[11] Yu D., Jing D., Yang F., Gao P., Jiang Q., Xu Y., Yu P., Xia W. (2021). The factors influencing the flavor characteristics of frozen obscure pufferfish (*Takifugu obscurus*) during storage: Ice crystals, endogenous proteolysis and oxidation. Int. J. Refrig..

[12] Wang H., Wang Y., Xu K., Pan S., Shi W., Wang X. (2024). Changes in water-soluble taste compounds of tilapia (*Oreochromis niloticus*) fillets subjected to different thawing methods during long-term frozen storage. J. Sci. Food Agric..

[13] Huang Y.-Z., Liu Y., Zhu R., Ma X., Xin S., Zhu B., Dong X.-P. (2024). Multi-omics Analysis of Volatile Flavor Components in Pacific Chub and Spanish Mackerel during Freezing using GC–MS–O. Food Chem..

[14] Fang Z., Zhong S., Li Q., Li C., He Y., Zhu L., Feng A., Hong H., Zhang L. (2026). Alternating magnetic field-assisted freezing enhances freeze-thaw quality of golden pompano (*Trachinotus ovatus*) fillets: Metabolomic and physicochemical insights from thawing exudate. Innov. Food Sci. Emerg. Technol..

[15] Teng X., Liu Y., Chen L., Xue C., Li Z. (2023). Effects of liquid nitrogen freezing at different temperatures on the quality and flavor of Pacific oyster (*Crassostrea gigas*). Food Chem..

[16] Hu Y., Quan Z., Wang Z., Luo Y., Guo X., Dong X., Zhou D., Zhu B. (2025). Uncovering quality changes in oysters (*Crassostrea hongkongensis*) during frozen storage based on lipidomics and proteomics. Food Chem..

[17] Chen L., Teng X., Liu Y., Shi H., Li Z., Xue C. (2024). The dynamic change of flavor characteristics in Pacific oyster (*Crassostrea gigas*) during depuration uncovered by mass spectrometry-based metabolomics combined with gas chromatography-ion mobility spectrometry (GC-IMS). Food Chem..

[18] Zhao N., Xu X., Dong S., Zhao Y., Liu T., Liu L., Zhao Y., Zeng M., Liu K. (2025). Effects of steam processing on the flavor formation mechanism of male and female mussels. LWT.

[19] Zhang M.-R., Ning Y.-F., Wang C.-C., Wang Y.-M., Abdelnaby T., Zhang T.-T. (2026). Lipid profile and fatty acid composition of the male and female *Mytilus edulis* from China. Food Med. Homol..

[20] Ji C., Wei L., Zhao J., Wu H. (2014). Metabolomic analysis revealed that female mussel *Mytilus galloprovincialis* was sensitive to bisphenol A exposures. Environ. Toxicol. Pharmacol..

[21] Xin Z., Wang H., Wei B., Qi P., Yan X., Liao Z., Guo B., Wang W. (2025). Gonadal Lipid Storage in *Mytilus coruscus*: A Comprehensive Gene Network and Key Gene Discovery. Mar. Biotechnol..

[22] Ma L.-X., Huang X.-H., Zheng J., Dong L., Chen J.-N., Dong X.-P., Zhou D.-Y., Zhu B.-W., Qin L. (2022). Free amino acid, 5′-Nucleotide, and lipid distribution in different tissues of blue mussel (*Mytilis edulis* L.) determined by mass spectrometry based metabolomics. Food Chem..

[23] James L., Gomez E., Ramirez G., Dumas T., Courant F. (2023). Liquid chromatography-mass spectrometry based metabolomics investigation of different tissues of *Mytilus galloprovincialis*. Comp. Biochem. Physiol. Part D Genom. Proteom..

[24] Martínez-Pita I., Sánchez Lazo C., Ruiz-Jarabo I., Herrera M., Mancera J. (2012). Biochemical composition, lipid classes, fatty acids and sexual hormones in the mussel *Mytilus galloprovincialis* from cultivated populations in South Spain. Aquaculture.

[25] Li R., Xiu X., Xiang J., Qu W., Chen L., Wang X., Jiang X., Liu H., Feng T., Xue C. (2026). Characterization of Species- and Sex-Dependent Volatile and Non-Volatile Flavor Profiles in Three Commercially Important Mussel Species. Foods.

[26] Laudicella V.A., Carboni S., Whitfield P.D., Doherty M.K., Hughes A.D. (2023). Sexual dimorphism in the gonad lipidome of blue mussels (*Mytilus* sp.): New insights from a global lipidomics approach. Comp. Biochem. Physiol. Part D Genom. Proteom..

[27] (2016). National Food Safety Standard—Determination of Taurine in Foods.

[28] Xing A., Sun Y., Wei F., Yang L., Zheng H., Xue C. (2025). Deep-learning-assisted chemo-responsive alizarin red S-based hydrogel sensor for the rapid freshness sensing of aquatic product. Food Res. Int..

[29] (2016). National Food Safety Standard—Determination of Fatty Acids in Foods.

[30] Abdelnaby T., Feng T., Zhang T., Jiang X., Wang Y., Li Z., Xue C. (2024). Impact of frozen storage on physicochemical parameters and quality changes in cooked crayfish. Heliyon.

[31] Chan S.S., Roth B., Skare M., Hernar M., Jessen F., Løvdal T., Jakobsen A.N., Lerfall J. (2020). Effect of chilling technologies on water holding properties and other quality parameters throughout the whole value chain: From whole fish to cold-smoked fillets of Atlantic salmon (*Salmo salar*). Aquaculture.

[32] Hematyar N., Rustad T., Sampels S., Dalsgaard T.K. (2019). Relationship between lipid and protein oxidation in fish. Aquac. Res..

[33] Wang Q., Lu S., Tao Y., Hua J., Zhuge Y., Chen W., Qiang J. (2024). Characteristic Muscle Quality Parameters of Male Largemouth Bass (*Micropterus salmoides*) Distinguished from Female and Physiological Variations Revealed by Transcriptome Profiling. Biology.

[34] Liu Z., Liu Q., Wei S., Sun Q., Xia Q., Zhang D., Shi W., Ji H., Liu S. (2021). Quality and volatile compound analysis of shrimp heads during different temperature storage. Food Chem. X.

[35] (2015). National Food Safety Standard—Fresh and Frozen Aquatic Products of Animal Origin.

[36] Bekhit A.E.-D.A., Holman B.W.B., Giteru S.G., Hopkins D.L. (2021). Total volatile basic nitrogen (TVB-N) and its role in meat spoilage: A review. Trends Food Sci. Technol..

[37] Baron C.P., Kristinsson H.G. (2014). Protein oxidation in aquatic foods. Antioxidants and Functional Components in Aquatic Foods.

[38] Yu Y.J., Yang S.P., Lin T., Qian Y.F., Xie J., Hu C. (2020). Effect of Cold Chain Logistic Interruptions on Lipid Oxidation and Volatile Organic Compounds of Salmon (*Salmo salar*) and Their Correlations with Water Dynamics. Front. Nutr..

[39] Dragoev S.G. (2024). Lipid Peroxidation in Muscle Foods: Impact on Quality, Safety and Human Health. Foods.

[40] Geng L., Liu K., Zhang H. (2023). Lipid oxidation in foods and its implications on proteins. Front. Nutr..

[41] Han J., Sun Y., Sun R., Zhang T., Wang C., Jiang N. (2022). Effects of freeze-thaw cycles on physicochemical properties and structure of cooked crayfish (*Procambarus clarkii*). Food Prod. Process. Nutr..

[42] Luo Y., Xu J., Fan Y.-C., Wang Z.-H., Hu Y.-Y., Li D.-Y., Liu Y.-X., Zhou D.-Y. (2025). Effect of lipid oxidation on the color deterioration of ready-to-eat oysters: Discussion based on the model system. Food Chem..

[43] Hamre K., Lie Ø., Sandnes K. (2003). Development of lipid oxidation and flesh colour in frozen stored fillets of Norwegian spring-spawning herring (*Clupea harengus* L.). Effects of treatment with ascorbic acid. Food Chem..

[44] Bonilla F., Reyes V., Chouljenko A., Dzandu B., Sathivel S. (2020). Influence of energy removal rate on the quality of minced meat from undersized crawfish during frozen storage. Food Prod. Process. Nutr..

[45] Shi L., Yang T., Xiong G., Li X., Wang X., Ding A., Qiao Y., Wu W., Liao L., Wang L. (2018). Influence of frozen storage temperature on the microstructures and physicochemical properties of pre-frozen perch (*Micropterus salmoides*). LWT.

[46] Li R., Sun Z., Zhao Y., Li L., Yang X., Chen S., Wei Y., Li C., Wang Y. (2022). Effect of different thermal processing methods on water-soluble taste substances of tilapia fillets. J. Food Compos. Anal..

[47] Rusanova P., Bono G., Dara M., Falco F., Gancitano V., Lo Brutto S., Okpala C.O.R., Nirmal N.P., Quattrocchi F., Sardo G. (2022). Effect of different packaging methods on the free amino acid profiles of the deep-water rose shrimp (*Parapenaeus longirostris*) during frozen storage. Front. Nutr..

[48] Lv Y., Xie J. (2021). Effects of Freeze-Thaw Cycles on Water Migration, Microstructure and Protein Oxidation in Cuttlefish. Foods.

[49] Suárez-Medina M.D., Sáez-Casado M.I., Martínez-Moya T., Rincón-Cervera M.Á. (2024). The Effect of Low Temperature Storage on the Lipid Quality of Fish, Either Alone or Combined with Alternative Preservation Technologies. Foods.

[50] Zhang H., Cui N., Ma X., Xu H.E. (2025). Structural basis of augmenting taurine uptake by the taurine transporter in alleviating cellular senescence. Cell Res..

[51] Zhuang S., Tian L., Liu Y., Wang L., Hong H., Luo Y. (2023). Amino acid degradation and related quality changes caused by common spoilage bacteria in chill-stored grass carp (*Ctenopharyngodon idella*). Food Chem..

[52] Wang Q., Xue C., Li Z., Fu X., Xu J., Xue Y. (2007). Changes in the contents of ATP and its related breakdown compounds in various tissues of oyster during frozen storage. J. Ocean Univ. China.

[53] Wei H., Tian Y., Lin Y., Maeda H., Yamashita T., Yu K., Takaki K., Yuan C. (2020). Condition-dependent adenosine monophosphate decomposition pathways in striated adductor muscle from Japanese scallop (*Patinopecten yessoensis*). J. Food Sci..

[54] Dong S., Niu Y., Wei H., Lin Y., Lu X., Yamashita T., Yu K., Takaki K., Yuan C. (2023). Effect of super-chilling storage on maintenance of quality and freshness of the Pacific oyster (*Crassostrea gigas*). Food Qual. Saf..

[55] Ma L., Lu J., Yao T., Ye L., Wang J. (2022). Gender-Specific Metabolic Responses of *Crassostrea hongkongensis* to Infection with *Vibrio harveyi* and Lipopolysaccharide. Antioxidants.

[56] Liu X., Sun H., Wang Y., Ma M., Zhang Y. (2014). Gender-specific metabolic responses in hepatopancreas of mussel *Mytilus galloprovincialis* challenged by *Vibrio harveyi*. Fish Shellfish Immunol..

[57] Zhan F., Wu Q., Zheng W., Pan D., Benjakul S., Liu Z., Lin H., Zhang B. (2025). Effects of DBD-CP pre-treatment on muscle quality, volatile flavor compounds, and microbial community composition of ready-to-eat drunken shrimp (*Solenocera crassicornis*) during frozen storage. Food Chem. X.

[58] Fu J., Sun Y., Cui M., Zhang E., Dong L., Wang Y., Wang W., Li Z., Yang J. (2023). Analysis of Volatile Compounds and Flavor Fingerprint Using Gas Chromatography-Ion Mobility Spectrometry (GC-IMS) on *Crassostrea gigas* with Different Ploidy and Gender. Molecules.

[59] Yang H., Khoder R.M., Xiong S., You J., An Y. (2026). Unraveling the link between lipid oxidation and off-flavor development in grass carp fillets (*Ctenopharyngodon idellus*) during cold storage: A non-targeted lipidomics approach. Food Sci. Hum. Wellness.

[60] Cheng H., Mei J., Xie J. (2023). Analysis of key volatile compounds and quality properties of tilapia (*Oreochromis mossambicus*) fillets during cold storage: Based on thermal desorption coupled with gas chromatography-mass spectrometry (TD-GC-MS). LWT.

