# Supplementary material for: Sex-Dependent Changes in Quality and Flavor-Related Chemical Profiles of Mytilus coruscus During Long-Term Frozen Storage
Source: Foods. 2026 Sep 21;15(18):3351. doi: 10.3390/foods15183351 (PMC13606257; doi:10.3390/foods15183351)
Supplement: Supplementary file 1 [file foods-15-03351-s001.zip › foods-4536505-supplementary.pdf]

Table S1

Changes in quality attributes, ATP-related compounds, and equivalent umami concentration of female and male mussels during frozen storage

	M0	M90	M150	F0	F90	F150
**Centrifugal loss** (%)	25.93 ± 0.5^{c}	31.39 ± 1.05^{b}	38.46 ± 0.77^{a}	25.61 ± 0.73^{C}	27.86 ± 0.69^{B}	29.7 ± 0.46^{A}
**TVB-N** (mg/100g)	3.49 ± 0.22^{b}	6.76 ± 0.24^{a}	8.34 ± 0.67^{a}	3.50 ± 0.05^{B}	9.14 ± 0.54^{A}	9.61 ± 0.35^{A}
**MDA** (nmol/mg prot)	2.89 ± 0.04^{c}	7.35 ± 0.42^{b}	9.65 ± 0.23^{a}	5.03 ± 1.10^{B}	15.8 ± 0.30^{A}	16.44 ± 0.99^{A}
**Hardenss** (N)	3.38 ± 0.20^{a}	0.69 ± 0.07^{b}	0.49 ± 0.07^{b}	2.85 ± 0.16^{A}	0.65 ± 0.17^{B}	0.40 ± 0.06^{B}
**Chewiness** (N)	5.47 ± 0.67^{a}	0.17 ± 0.06^{b}	0.05 ± 0.01^{b}	3.77 ± 0.35^{A}	0.10 ± 0.10^{B}	0.05 ± 0.01^{B}
**ΔE**	-	10.97 ± 2.07^{b}	15.07 ± 1.40 ^{a}	-	32.66 ± 2.59^{A}	34.03 ± 0.82^{A}
**ATP** (mg/100g)	27.13 ± 3.34^{a}	20.59 ± 1.19^{b}	17.05 ± 0.18^{b}	52.13 ± 5.27^{A}	21.14 ± 0.80^{B}	16.52 ± 1.02^{B}
**ADP** (mg/100g)	19.58 ± 2.95^{a}	10.87 ± 0.19^{a}	10.40 ± 0.50^{a}	31.69 ± 0.67^{A}	11.71 ± 0.91^{B}	11.58 ± 2.63^{B}
**AMP** (mg/100g)	18.89 ± 2.37^{c}	28.23 ± 1.24^{a}	23.03 ± 1.53^{b}	16.89 ± 1.50^{B}	26.12 ± 1.74^{A}	24.57 ± 4.74^{A}
**IMP** (mg/100g)	5.6 ± 0.98^{a}	6.06 ± 0.07^{a}	6.17 ± 0.42^{a}	7.59 ± 1.35^{B}	14.78 ± 0.68^{A}	9.80 ± 5.27^{AB}
**GMP** (mg/100g)	3.37 ± 1.01^{b}	7.58 ± 0.21^{a}	8.24 ± 0.38^{a}	2.91 ± 0.15^{C}	12.14 ± 1.15^{A}	10.11 ± 0.82^{B}
**Hx** (mg/100g)	0.97 ± 0.17^{c}	1.94 ± 0.07^{a}	1.57 ± 0.11^{b}	0.45 ± 0.01^{B}	0.74 ± 0.05^{A}	0.70 ± 0.02^{A}
**HxR** (mg/100g)	0.64 ± 0.01^{c}	5.73 ± 0.49^{b}	11.44 ± 0.79^{a}	0.63 ± 0.01^{B}	13.89 ± 2.16^{A}	14.49 ± 2.49^{A}
**EUC** (g MSG/100 g)	2.24 ± 0.56^{b}	5.38 ± 0.15^{a}	5.85 ± 0.45^{a}	2.00 ± 0.17^{B}	7.51 ± 0.09^{AB}	5.74 ± 1.02^{B}

Note: Concentration data are presented as mean ± SD. Different superscript lowercase letters (a, b, c) denote significant differences among males ($p < 0.05$), while different superscript uppercase letters (A, B, C) denote significant differences among females ($p < 0.05$). F0 and M0 were used as the respective references for ΔE calculation; therefore, their ΔE values (zero by definition) are indicated by "-".

Table S2

Changes in free amino acids of female and male mussels during frozen storage (**mg/100 g**)

	M0	M90	M150	F0	F90	F150
**Asp**	31.79 ± 6.25^{a}	39.82 ± 4.58^{a}	34.05 ± 2.68^{a}	36.24 ± 1.18^{A}	36.49 ± 1.98^{A}	38.27 ± 4.11^{A}
**Glu**	101.55 ± 8.10^{b}	147.25 ± 2.87^{a}	157.02 ± 6.3^{a}	87.63 ± 4.02^{B}	125.42 ± 8.26^{A}	120.04 ± 4.71^{A}
**Ser**	33.02 ± 1.63^{b}	48.12 ± 2.89^{a}	48.53 ± 2.26^{a}	34.05 ± 1.33^{C}	53.71 ± 0.97^{A}	47.48 ± 3.43^{B}
**Thr**	8.01 ± 0.56^{b}	12.55 ± 1.09^{a}	13.04 ± 0.66^{a}	7.25 ± 0.27^{B}	12.30 ± 0.68^{A}	11.11 ± 0.95^{A}
**Ala**	49.94 ± 7.22^{b}	81.86 ± 2.54^{a}	85.49 ± 3.86^{a}	59.52 ± 0.56^{C}	91.19 ± 1.36^{A}	88.52 ± 1.40^{B}
**Pro**	11.35 ± 0.34^{b}	16.89 ± 0.48^{a}	17.40 ± 0.93^{a}	10.68 ± 0.72^{B}	17.59 ± 1.51^{A}	16.22 ± 1.32^{A}
**Gly**	214.79 ± 20.84^{b}	279.89 ± 18.22^{a}	294.33 ± 3.93^{a}	141.99 ± 10.48^{B}	186.20 ± 10.02^{A}	187.48 ± 13.63^{A}
**His**	9.79 ± 2.21^{a}	12.34 ± 1.68^{a}	12.12 ± 0.78^{a}	7.02 ± 0.76^{A}	8.02 ± 1.06^{A}	7.95 ± 0.84^{A}
**Arg**	65.19 ± 6.64^{b}	80.48 ± 1.90^{a}	82.56 ± 2.48^{a}	58.18 ± 5.83^{A}	59.19 ± 3.26^{A}	57.97 ± 3.14^{A}
**Met**	6.00 ± 1.06^{b}	7.64 ± 0.54^{a}	7.09 ± 0.60^{ab}	6.57 ± 0.24^{A}	7.02 ± 0.58^{A}	6.18 ± 1.25^{A}
**Ile**	6.08 ± 0.12^{b}	8.97 ± 0.56^{a}	9.99 ± 0.82^{a}	6.03 ± 0.37^{C}	10.55 ± 0.42^{A}	9.18 ± 0.29^{B}
**Leu**	9.76 ± 0.16^{b}	14.81 ± 1.52^{a}	15.41 ± 0.91^{a}	9.49 ± 0.52^{C}	16.24 ± 0.55^{A}	13.70 ± 0.63^{B}
**Lys**	14.17 ± 3.59^{b}	27.00 ± 4.41^{a}	31.16 ± 1.54^{a}	11.21 ± 0.60^{B}	17.31 ± 1.99^{AB}	15.17 ± 0.69^{A}
**Phe**	5.77 ± 0.16^{b}	9.06 ± 0.88^{a}	8.33 ± 0.54^{a}	6.37 ± 0.36^{B}	8.21 ± 0.66^{A}	8.31 ± 0.16^{A}
**Val**	8.20 ± 0.18^{c}	12.21 ± 1.16^{b}	13.97 ± 0.49^{a}	8.32 ± 0.35^{C}	14.27 ± 0.49^{A}	12.62 ± 0.39^{B}
**Tyr**	17.21 ± 3.23^{a}	22.31 ± 2.98^{a}	21.59 ± 2.17^{a}	14.80 ± 0.52^{B}	21.70 ± 1.07^{A}	20.78 ± 2.30^{A}
**UAAs**	133.34 ± 7.98^{a}	187.07 ± 6.19^{a}	191.07 ± 8.12^{b}	123.87 ± 3.91^{B}	161.91 ± 9.08^{A}	158.31 ± 4.64^{A}
**SAAs**	317.11 ± 16.98^{a}	439.31 ± 15.7^{a}	458.78 ± 1.36^{b}	253.49 ± 12.34^{B}	360.99 ± 8.85^{A}	350.80 ± 10.66^{A}
**BAAs**	110.99 ± 6.73^{a}	151.25 ± 9.69^{a}	158.32 ± 5.65^{b}	98.51 ± 7.39^{B}	118.32 ± 2.71^{A}	110.16 ± 2.95^{A}
**TFAAs**	592.62 ± 30.08^{a}	821.21 ± 19.18^{a}	852.07 ± 13.93^{b}	505.36 ± 22.41^{B}	685.40 ± 17.91^{A}	660.99 ± 8.52^{A}

Note: Concentration data are presented as mean ± SD. Different superscript lowercase letters (a, b, c) denote significant differences among males ($p < 0.05$), while different superscript uppercase letters (A, B, C) denote significant differences among females ($p < 0.05$). The full names and abbreviations of amino acids are as follows. Aspartic acid (Asp), Glutamine (Glu), Serine (Ser), Glycine (Gly), Histidine(His), Arginine (Arg), Threonine (Thr), Alanine (Ala), Proline (Pro), Valine (Val), Methionine (Met), Isoleucine (Ile), Leucine (Leu), Lysine (Lys), Tyrosine (Tyr), Phenylalanine (Phe), Taurine (Tau), TFAAs= total free amino acids. Umami amino acids (UAAs): Asp+Glu ;Sweet amino acids (SAAs): Ser+Thr+Ala+Pro+Gly; Bitter amino acids (BAAs): His+Arg+Met+Ile+Leu+Lys.

Table S3

Changes in fatty acid composition of female and male mussels during frozen storage (% of total fatty acids)

	M0	M90	M150	F0	F90	F150
**C14:0**	1.29 ± 0.04^{b}	1.43 ± 0.04^{a}	1.53 ± 0.07^{a}	2.79 ± 0.05^{A}	3.11 ± 0.22^{A}	2.89 ± 0.21^{A}
**C15:0**	0.56 ± 0.03^{b}	0.62 ± 0.04^{ab}	0.67 ± 0.03^{a}	0.68 ± 0.02^{A}	0.73 ± 0.04^{A}	0.73 ± 0.02^{A}
**C16:0**	24.51 ± 0.05^{b}	26.39 ± 0.66^{a}	26.89 ± 0.26^{a}	26.39 ± 0.21^{B}	27.58 ± 0.35^{A}	28.12 ± 0.44^{A}
**C17:0**	2.30 ± 0.03^{a}	2.37 ± 0.16^{a}	2.41 ± 0.06^{a}	1.30 ± 0.05^{B}	1.34 ± 0.04^{B}	1.46 ± 0.07^{A}
**C18:0**	6.61 ± 0.10^{a}	6.87 ± 0.45^{a}	6.97 ± 0.28^{a}	4.37 ± 0.10^{B}	4.42 ± 0.18^{B}	4.89 ± 0.32^{A}
**C20:0**	0.25 ± 0.02^{b}	0.31 ± 0.03^{a}	0.30 ± 0.01^{a}	0.48 ± 0.05^{A}	0.49 ± 0.05^{A}	0.52 ± 0.04^{A}
**C24:0**	0.55 ± 0.01^{a}	0.55 ± 0.03^{a}	0.55 ± 0.01^{a}	0.34 ± 0.02^{C}	0.43 ± 0.03^{B}	0.50 ± 0.02^{A}
**SFA**	36.08 ± 0.07^{b}	38.55 ± 1.28^{ab}	39.30 ± 0.27^{a}	36.36 ± 0.18^{C}	38.09 ± 0.52^{B}	39.12 ± 0.56^{A}
**C16:1**	3.70 ± 0.15^{b}	4.31 ± 0.05^{a}	4.41 ± 0.28^{ab}	8.54 ± 0.17^{A}	9.45 ± 0.58^{A}	8.63 ± 0.18^{A}
**C17:1**	0.13 ± 0.02^{a}	0.14 ± 0.00^{a}	0.14 ± 0.00^{a}	0.28 ± 0.02^{A}	0.26 ± 0.04^{A}	0.26 ± 0.01^{A}
**C18:1n9t**	0.28 ± 0.02^{a}	0.28 ± 0.02^{a}	0.31 ± 0.01^{a}	0.27 ± 0.01^{B}	0.31 ± 0.03^{A}	0.33 ± 0.01^{A}
**C18:1n9c**	1.81 ± 0.07^{a}	1.99 ± 0.21^{a}	2.19 ± 0.24^{a}	2.91 ± 0.11^{A}	3.12 ± 0.21^{A}	3.05 ± 0.09^{A}
**C20:1**	4.79 ± 0.04^{a}	5.02 ± 0.33^{a}	4.93 ± 0.14^{a}	3.61 ± 0.11^{A}	3.72 ± 0.30^{A}	3.93 ± 0.18^{A}
**C22:1**	0.68 ± 0.06^{a}	0.73 ± 0.07^{a}	0.62 ± 0.07^{a}	0.59 ± 0.05^{A}	0.63 ± 0.04^{A}	0.6 ± 0.06^{A}
**MUFA**	11.38 ± 0.18^{b}	12.47 ± 0.58^{a}	12.59 ± 0.30^{a}	16.19 ± 0.02^{A}	17.49 ± 0.89^{A}	16.8 ± 0.20^{A}
**C18:2n6c**	1.62 ± 0.02^{b}	1.72 ± 0.10^{ab}	1.78 ± 0.07^{a}	1.79 ± 0.08^{A}	1.82 ± 0.05^{A}	1.85 ± 0.02^{A}
**C18:3n6**	0.09 ± 0.01^{a}	0.10 ± 0.01^{a}	0.09 ± 0.01^{a}	0.08 ± 0.01^{C}	0.11 ± 0.01^{B}	0.13 ± 0.01^{A}
**C18:3n3**	1.42 ± 0.05^{a}	1.25 ± 0.13^{a}	1.39 ± 0.07^{a}	1.77 ± 0.09^{A}	1.74 ± 0.17^{A}	1.68 ± 0.06^{A}
**C20:2**	0.54 ± 0.01^{a}	0.55 ± 0.01^{a}	0.52 ± 0.06^{a}	0.52 ± 0.04^{A}	0.54 ± 0.03^{A}	0.56 ± 0.04^{A}
**C20:3n6**	0.20 ± 0.02^{a}	0.19 ± 0.01^{a}	0.17 ± 0.02^{a}	0.17 ± 0.02^{A}	0.17 ± 0.01^{A}	0.17 ± 0.02^{A}
**C20:4n6**	4.13 ± 0.16^{a}	3.80 ± 0.20^{a}	3.82 ± 0.14^{a}	3.02 ± 0.18^{A}	2.88 ± 0.27^{A}	3.08 ± 0.08^{A}
**C20:5n3**	22.39 ± 0.26^{a}	20.57 ± 1.04^{b}	20.12 ± 0.12^{b}	20.18 ± 0.39^{A}	18.77 ± 0.74^{B}	18.38 ± 0.25^{B}
**C22:6n3**	22.14 ± 0.35^{a}	20.81 ± 0.72^{b}	20.22 ± 0.18^{b}	19.9 ± 0.13^{A}	18.38 ± 0.50^{B}	18.24 ± 0.51^{B}
**PUFA**	52.54 ± 0.20^{b}	48.98 ± 1.78^{ab}	48.11 ± 0.09^{a}	47.44 ± 0.20^{B}	44.41 ± 1.26^{B}	44.08 ± 0.74^{A}
**ω3/ω6**	7.61 ± 0.19^{a}	7.35 ± 0.39^{b}	7.12 ± 0.07^{b}	8.29 ± 0.48^{A}	7.83 ± 0.53^{AB}	7.32 ± 0.11^{B}

Note: Concentration data are presented as mean ± SD. Different superscript lowercase letters (a, b, c) denote significant differences among males ($p < 0.05$), while different superscript uppercase letters (A, B, C) denote significant differences among females ($p < 0.05$).

Table S4

Differences in volatile flavor compounds between male and female mussels

Chemical class	Compound	CAS#	Formula	MW	RI	Rt [sec]	Dt [a.u.]
Aldehydes	Benzaldehyde-M	100-52-7	C7H6O	106.1	1509.7	968.092	1.15365
	Benzaldehyde-D	100-52-7	C7H6O	106.1	1509.9	968.582	1.47113
	1-octanal-M	124-13-0	C8H16O	128.2	1297.5	608.002	1.40636
	1-octanal-D	124-13-0	C8H16O	128.2	1297.3	607.659	1.81666
	1-nonanal	124-19-6	C9H18O	142.2	1394.3	761.529	1.47343
	(Z)-4-heptenal	6728-31-0	C7H12O	112.2	1244.2	517.3	1.14835
	(E)-2-hexen-1-al-M	6728-26-3	C6H10O	98.1	1219.7	479.833	1.17939
	(E)-2-hexen-1-al-D	6728-26-3	C6H10O	98.1	1217.9	477.049	1.51557
	Heptaldehyde-M	111-71-7	C7H14O	114.2	1180.4	424.896	1.33519
	Heptaldehyde-D	111-71-7	C7H14O	114.2	1181	425.751	1.69038
	(E)-2-Pentenal-D	1576-87-0	C5H8O	84.1	1131.3	357.287	1.35716
	(E)-2-Pentenal-M	1576-87-0	C5H8O	84.1	1131	357.014	1.10563
	1-hexanal-M	66-25-1	C6H12O	100.2	1083.9	302.747	1.26915
	1-hexanal-D	66-25-1	C6H12O	100.2	1085	303.847	1.55929
	2-Ethyl butanal	97-96-1	C6H12O	100.2	997.6	231.501	1.50612
	Butanal	123-72-8	C4H8O	72.1	906.8	173.407	1.28803
	Propanal	123-38-6	C3H6O	58.1	822.3	132.435	1.13893
Alcohols	1-Heptanol	111-70-6	C7H16O	116.2	1463	877.02	1.40173
	1-Hexanol-M	111-27-3	C6H14O	102.2	1366.4	713.822	1.3269
	(Z)-2-pentenol	1576-95-0	C5H10O	86.1	1337.8	667.813	1.44609
	(Z)-3-hexen-1-ol	928-96-1	C6H12O	100.2	1391.2	756.38	1.23462
	2-Heptanol	543-49-7	C7H16O	116.2	1337.7	667.709	1.37392
	1-Hexanol-D	111-27-3	C6H14O	102.2	1365.9	712.973	1.64193
	1-Pentanol-M	71-41-0	C5H12O	88.1	1255.2	535.199	1.25363
	1-Pentanol-D	71-41-0	C5H12O	88.1	1254.5	534.089	1.51134
	1-Butanol	71-36-3	C4H10O	74.1	1156.7	390.837	1.38403
	1-Propanol	71-23-8	C3H8O	60.1	1040.3	264.346	1.11536
	Ethanol-M	64-17-5	C2H6O	46.1	956.7	203.347	1.04365
	Ethanol-D	64-17-5	C2H6O	46.1	939.8	192.62	1.12889
	2-Propanol	67-63-0	C3H8O	60.1	942.7	194.472	1.22427
	2-Methyl-2-propanol	75-65-0	C4H10O	74.1	925.9	184.287	1.32669
Ketones	3-Methyl-2-cyclopenten-1-one	2758-18-1	C6H8O	96.1	1498.3	943.611	1.10855
	2-methyl-2-hepten-6-one	110-93-0	C8H14O	126.2	1347.9	683.729	1.17469
	2-Nonanone	821-55-6	C9H18O	142.2	1389.1	752.642	1.40707
	5-Methyl-3-heptanone	541-85-5	C8H16O	128.2	1274.5	568.003	1.27461
	2-Heptanone	110-43-0	C7H14O	114.2	1175	416.793	1.26187
	1-Penten-3-one-D	1629-58-9	C5H8O	84.1	1024.5	251.65	1.31282
	1-Penten-3-one-M	1629-58-9	C5H8O	84.1	1025.5	252.478	1.07795
	4-Methyl-2-pentanone	108-10-1	C6H12O	100.2	1009.7	240.333	1.17668
	3-Pentanone-D	96-22-0	C5H10O	86.1	984	221.84	1.3575
	3-Pentanone-M	96-22-0	C5H10O	86.1	982.9	221.012	1.1112
	2-Butanone	78-93-3	C4H8O	72.1	914.2	177.574	1.24486
	Acetone	67-64-1	C3H6O	58.1	849.6	144.472	1.11483
Esters	Ethyl 2-hydroxypropanoate	97-64-3	C5H10O3	118.1	1342.9	675.865	1.14875
	Ethyl 3-ethoxypropanoate	763-69-9	C7H14O3	146.2	1336.6	665.914	1.22791
	Butyl 2-methylbutanoate	15706-73-7	C9H18O2	158.2	1258.3	540.277	1.37807
	Isopropyl butanoate	638-11-9	C7H14O2	130.2	1034.5	259.654	1.2567
	2-Methyl-1-propyl acetate	110-19-0	C6H12O2	116.2	1009.3	240.057	1.23383

Chemical class	Compound	CAS#	Formula	MW	RI	Rt [sec]	Dt [a.u.]
	sec-butyl acetate	105-46-4	C6H12O2	116.2	997.6	231.501	1.21201
	Methyl butanoate	623-42-7	C5H10O2	102.1	995.7	230.12	1.43337
	Ethyl acetate	141-78-6	C4H8O2	88.1	897.5	168.314	1.33522
	Methyl isovalerate	556-24-1	C6H12O2	116.2	1046.2	269.24	1.19867
	Methyl methacrylate	80-62-6	C5H8O2	100.1	1010.9	241.231	1.10881
Furans	Furan, 2-methyl-3-(methylthio)	63012-97-5	C6H8OS	128.2	1349.3	686.006	1.10254
	2-Pentylfuran	3777-69-3	C9H14O	138.2	1228.9	493.488	1.24892
	2-Butylfuran	4466-24-4	C8H12O	124.2	1142.8	372.07	1.18288
Sulfur-containin g compounds	Allyl isothiocyanate	57-06-7	C4H5NS	99.2	1366.1	713.31	1.09124
	Methyl thiocyanate	556-64-9	C2H3NS	73.1	1263.8	549.489	1.17624
	Diethyl disulfide	110-81-6	C4H10S2	122.2	1218.1	477.377	1.13163
Acids	Acetic acid-M	64-19-7	C2H4O2	60.1	1468.7	887.302	1.05477
	Acetic acid-D	64-19-7	C2H4O2	60.1	1469	887.792	1.15712
Nitrogen-contai ning compounds	Pyridine	110-86-1	C5H5N	79.1	1205.7	459.475	1.24444
	Triethylamine	121-44-8	C6H15N	101.2	794.8	121.324	1.08521
Heterocyclic compounds	Thiazole, 4-methyl-2-isopropyl	15679-13-7	C7H11NS	141.2	1338	668.196	1.62807
Hydrocarbons	2,4-Dimethyl-1-heptene	19549-87-2	C9H18	126.2	885.6	162.064	1.19817
Unidentified	1	unidentified	*	0	1312.9	630.239	1.22309
	2	unidentified	*	0	1405.9	779.938	1.26237
	3	unidentified	*	0	1288.4	592.926	1.10916
	4	unidentified	*	0	1239.1	509.229	1.12043
	5	unidentified	*	0	1221.3	482.15	1.40241
	6	unidentified	*	0	1113.2	335.291	1.08854
	7	unidentified	*	0	1097.6	317.344	1.09561
	8	unidentified	*	0	1097	316.682	1.34919
	9	unidentified	*	0	1019.8	248.061	1.14238
	10	unidentified	*	0	1012.3	242.265	1.03222
	11	unidentified	*	0	1046.6	269.591	1.0395
	12	unidentified	*	0	978.9	218.252	1.53833
	13	unidentified	*	0	964.7	208.591	1.70045
	14	unidentified	*	0	942	194.009	1.37137
	15	unidentified	*	0	890	164.379	1.27899
	16	unidentified	*	0	996	230.351	1.31163
